# The experience of body image in people with psychosis and psychotic‐like experiences: A co‐produced mixed‐methods systematic review and narrative synthesis

**DOI:** 10.1111/papt.70021

**Published:** 2025-11-17

**Authors:** Jenna McAllister, Sophie M. Allan, Alie Phiri, Kara Keddie, Tracey McKee, Leonie Richardson, Felicity Waite, Rebekah Carney, Gillian MacAfee, Andrew Gumley, Stephanie Allan

**Affiliations:** ^1^ School of Health & Wellbeing University of Glasgow Glasgow UK; ^2^ NHS Greater Glasgow & Clyde Glasgow UK; ^3^ Department of Clinical Psychology & Psychological Therapies, Medical School University of East Anglia Norwich UK; ^4^ Faculty of Health Sciences University of East Anglia Norwich UK; ^5^ Department of Experimental Psychology University of Oxford Oxford UK; ^6^ Oxford Health NHS Foundation Trust Oxford UK; ^7^ Greater Manchester Mental Health NHS Foundation Trust Manchester UK; ^8^ University of Manchester Manchester UK

**Keywords:** body dysmorphic disorder, psychosis, schizophrenia

## Abstract

**Purpose:**

Body image is a transdiagnostic construct that seems poorly understood in psychosis. Poor body image is associated with paranoia, which makes it a theoretically meaningful treatment target in psychosis. We systematically reviewed associations between body image and psychosis symptoms in both the ‘general’ population and people living with psychotic disorders, synthesised known correlates of negative body image in people living with psychotic disorders and performed a meta‐synthesis to understand the lived experience of body image in people with psychosis.

**Methods:**

Ovid MEDLINE, OVID Embase, OVID APA PsycINFO, EBSCOhost Cinahl and the Cochrane Central Register of Controlled Trials were searched in January 2024. The methodological quality and risk of bias were assessed using the mixed‐methods appraisal tool.

**Results:**

20,565 participants were included from 31 studies, of which 2127 (10.3%) were living with psychotic conditions, 18,294 from the general population, 129 people with other conditions being compared to psychosis (such as bipolar disorder) and 15 carers. There were 25 quantitative studies (24 cross‐sectional, 1 prospective), 5 qualitative studies and 1 mixed‐methods study. Cross‐sectional evidence suggests associations between negative body image and psychotic symptoms, especially paranoia, as well as wider mental and physical health outcomes. Potential factors contributing to the persistence of poor body image include psychotic symptoms, worries about appearance‐related judgements, negative self‐concept, body ambivalence, appearance‐related safety‐seeking behaviours and traumatic memories.

**Conclusions:**

Negative body image is relevant to the lives of people with psychosis spectrum conditions. Recommendations to guide and improve future research are reported.

## INTRODUCTION

Psychotic conditions are associated with profound physical and mental health problems. While statistics about the increased incidence of physical health problems in psychotic conditions, such as obesity (Firth et al., 2019), are widely known, there is less focus within the literature on what people with psychosis think and feel about their physical appearance and bodies. As far back as the 19th century, Kraeplin and Bleuler noted disturbances of body experience, such as seeing a distorted face, were common in people who would now be diagnosed with schizophrenia (Sakson‐Obada et al., 2018). Historically, these were considered to be aligned with perceptual disturbances linked to psychosis symptoms (Torregrossa et al., 2024), and not the modern transdiagnostic conceptualisation of body image, which is experienced in addition to symptoms.

### Body image

Body image is a multidimensional concept covering the subjective experience someone has about their physical body, which has cognitive (thoughts and beliefs about the body), affective (emotions related to the body) and behavioural components (Cash, 2012) related to factors such as appearance or bodily functioning, like the ability to be physically active. Body image is a crucial component of self‐concept, which is what one thinks about oneself. Negative body image refers to negative thoughts and emotions about the body, which may be linked with a desire to change one's appearance. Positive body image is a distinct construct from negative body image and describes appreciating and accepting one's appearance and/or bodily function (Tylka & Wood‐Barcalow, 2015b). Reducing negative body image is considered an important intervention for the maintenance of eating disorders (Levine & Smolak, 2018), and in improving the lives of people living with physical health conditions (Gillen, 2015) and autism (Longhurst, 2023), but no reviews to date have explored body image in the context of psychosis.

Holding negative views towards oneself and one's body may lead to a sense of inferiority and repetitive negative thinking. This negative self‐belief can motivate a meaning‐making process which results in paranoia. Paranoia at its most extreme can take the form of paranoid delusions (Marshall et al., 2020) which makes negative body image a theoretically meaningful treatment target in psychosis. Understanding body image in psychosis is important, given that negative body image may contribute to the occurrence of depression (Paans et al., 2018), anxiety (Barnes et al., 2020), and is associated with obesity (Weinberger et al., 2017) which can be a common antipsychotic medication side effect. Understanding how negative body image is experienced and manifested in the context of psychosis is critical for the development of psychological therapies. Understanding how positive body image is experienced in the psychosis context would be essential to understand how to promote positive body image in this population. However, because positive body image has been overlooked in body image research generally (Rodgers et al., 2023), it is expected not to be a major focus in existing research on psychosis.

Psychological therapies work by identifying and targeting what underpins a presenting problem. Therefore, there is a need to develop an understanding of what may precipitate or perpetuate body image concerns in this population and if there are any links with psychotic symptoms. Psychotic symptoms are commonly associated with conditions such as schizophrenia, but evidence suggests psychotic experiences exist upon a continuum (Van Os, 2016). The psychosis continuum encompasses a full range of psychotic symptom expressions from subclinical manifestations to clinically significant psychotic symptoms typically observed in individuals diagnosed with psychiatric conditions such as schizophrenia (Derosse & Karlsgodt, 2015). Therefore, there is merit in focusing on relationships between body image and psychotic‐like experiences in the general population as well as those with psychotic disorders. Understanding and addressing body image in the context of psychosis is not only important for promoting positive body image or potentially reducing paranoia, but also for its broader relevance to psychological well‐being. Interventions targeting body image may offer transdiagnostic benefits by supporting improvements in mood, enhancing emotional regulation and reducing behaviours that may compromise health and well‐being like eating pathologies (Linardon et al., 2022).

In this review we set out to (i) examine the relationship between body image and psychosis symptoms, (ii) explore body image from the lived experience of people with psychotic disorders, (iii) consider implications for further research and practice and (iv) synthesise correlates.

## METHODS

This review is reported in line with the Preferred Reporting Items for Systematic Review (PRISMA) guidelines (Page et al., 2021).

### Protocol and registration

The review protocol was developed in full collaboration with people with personal experience of the psychosis spectrum. The PROSPERO protocol (CRD42023407023) was registered on 10th March 2023; see here: https://www.crd.york.ac.uk/PROSPERO/view/CRD42023407023.

### Information sources and search strategy

The search strategy was designed by a health librarian (TM) with previous systematic review experience in collaboration with the review team. The review team included people with personal and academic expertise in body image in psychosis. The final draft search strategy was peer‐reviewed by a second health librarian external to the review using the PRESS checklist, which is a structured tool for peer review of electronic literature search strategies (McGowan et al., 2016). The search was initially conducted on 16th March 2023 across the following five databases from inception to last update: Ovid MEDLINE(R) ALL (1946 to March 15, 2023), OVID Embase Classic+Embase (1947 to 2023 March 15), OVID APA PsycInfo (1806 to March Week 1 2023), EBSCOhost Cinahl (1981 to last update) and the Cochrane Central Register of Controlled Trials (Issue 2 of 12, March 2023). The search was re‐run to identify new articles published between 15th March 2023 and 16th January 2024.

The search used a combination of database‐specific subject headings and keywords relating to the concepts ‘psychosis’ and ‘body image’. The strategy was adapted for each database to take account of differences in subject headings and other functionalities. The strategy was restricted to English‐language publications as the review team did not have access to funding for translation services. The full search strategies for each database can be found in Supplementary Appendix A. A backwards citation search was completed for all included studies.

**FIGURE 1:**
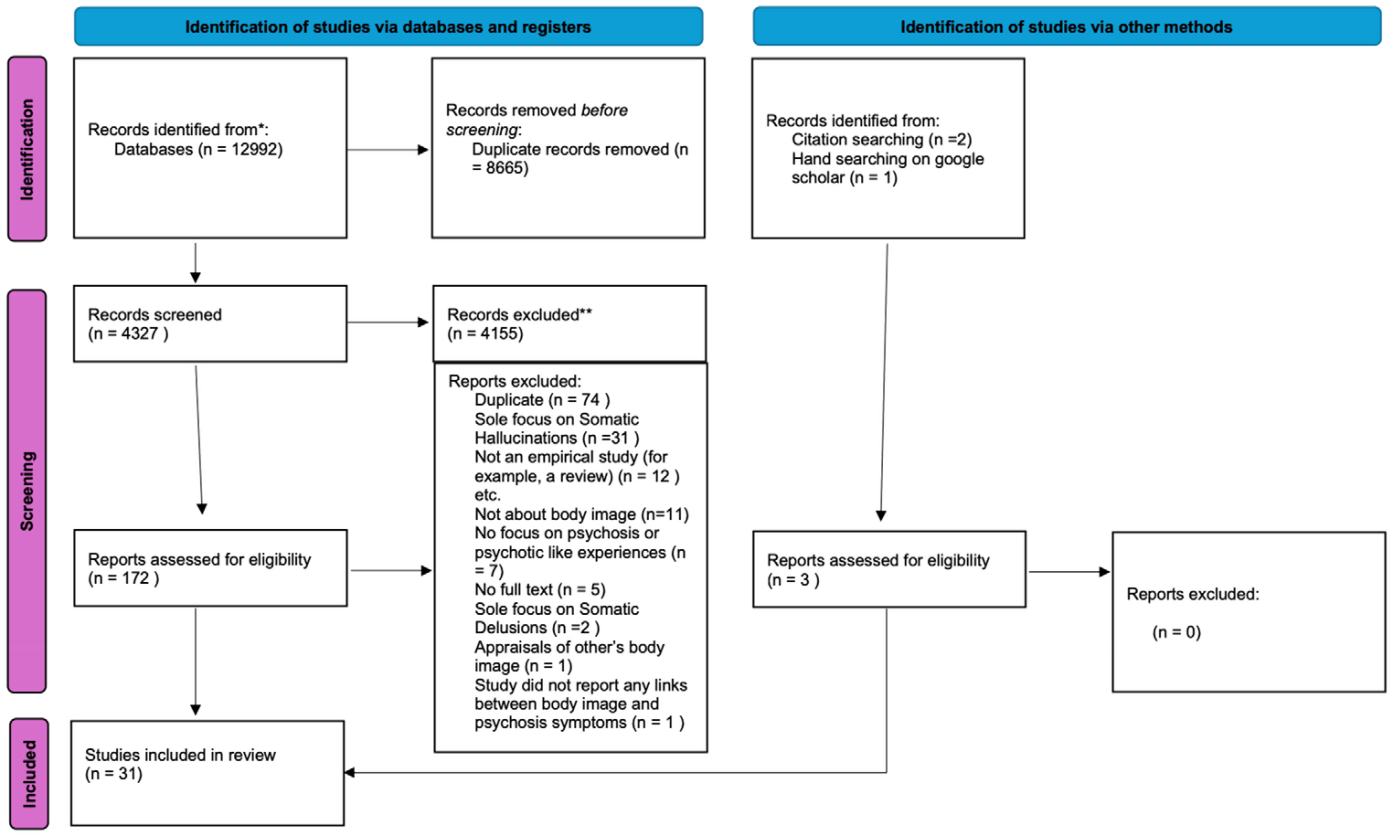
PRISMA flowchart.

A total of 12,992 citations were retrieved from the search methods as described above; the PRISMA diagram in Figure 1 details the flow of information throughout the review. The review team used Rayyan software (https://www.rayyan.ai/) for de‐duplication, screening and article management.

### Eligibility criteria

We included studies involving people diagnosed with psychotic conditions (diagnosis of affective and non‐affective psychosis spectrum conditions, including first episode psychosis), and also from ‘the general population’ including people experiencing attenuated and milder forms of psychotic‐like experiences (e.g., delusional‐like beliefs) (Aunjitsakul et al., 2021) which usually psychotic‐like experiences are assessed with continuum‐based scales. We included studies which explored body image (what one thinks and feels towards their body) using quantitative, qualitative or mixed methods.

### Study selection

The database results were combined, which resulted in 12,558 records. Following the removal of duplicates, all the titles of 4327 were screened independently by SA and JM, resulting in 172 records available for full‐text screening, which were all independently screened by SA and JM. After comparing to the inclusion criteria, 28 studies were available for narrative synthesis (Cohen Kappa = .86, original agreement between raters—disagreements resolved by consensus discussion). A further two were found by forward citation searching, and a further one by hand searching on Google Scholar, with agreement between SA and JM for inclusion of 100%.

### Quality assessment

Study quality was assessed using the mixed‐methods appraisal tool (MMAT) (Hong et al., 2018), which is a widely used tool designed for systematic reviews appraising the methodological quality of studies that use quantitative, qualitative and mixed‐methods designs to allow comment on the overall methodological rigour.

### Data synthesis

In line with Hong and colleagues (2017), this mixed‐methods review followed a results‐based convergent synthesis design. Qualitative and quantitative evidence were analysed separately using different synthesis methods, with the results of both syntheses integrated.

#### Qualitative analysis

**FIGURE 2:**
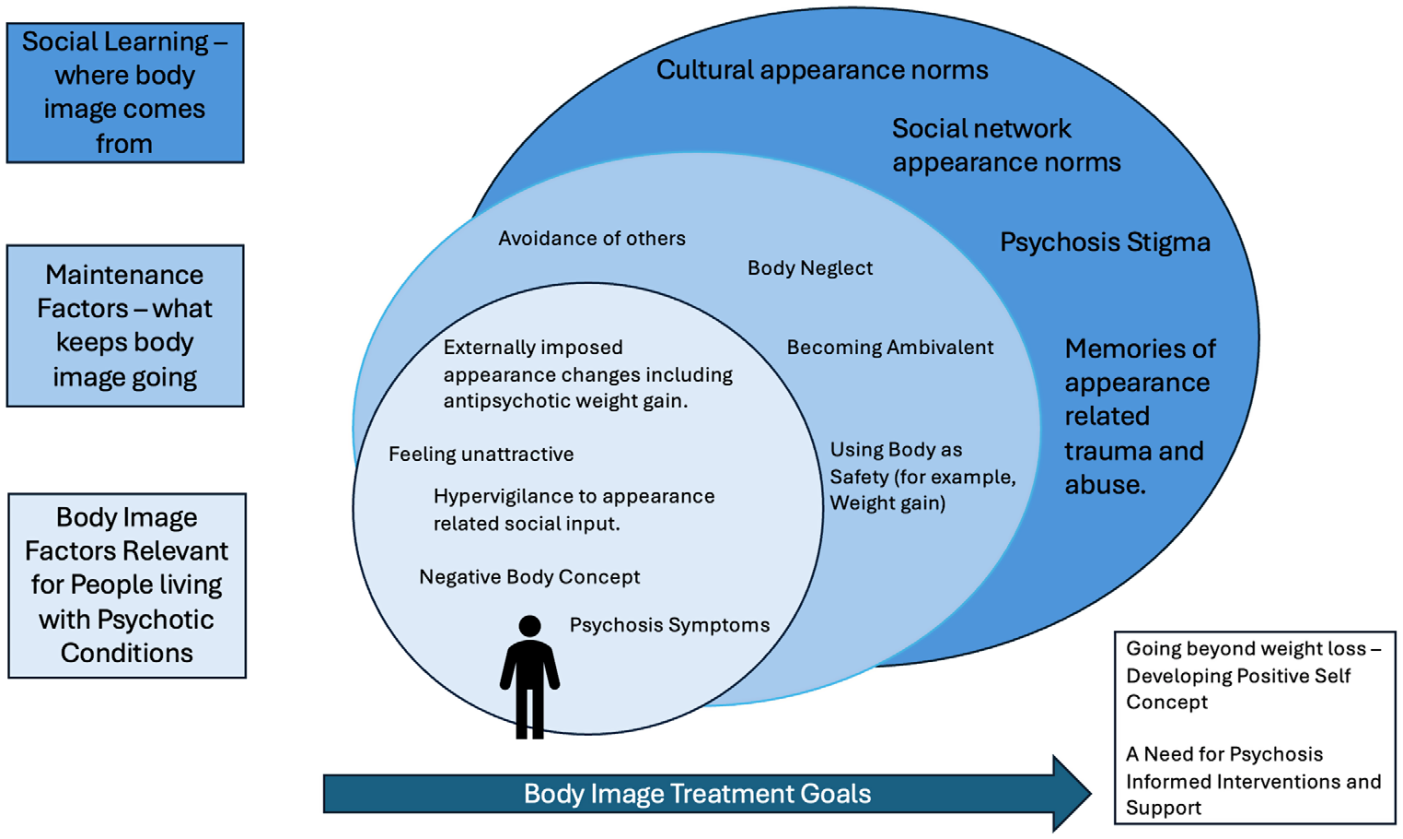
Results from meta‐synthesis.

Thomas and Harden's formal approach to thematic synthesis (Thomas & Harden, 2008) was used. This consists of three stages: (1) line‐by‐line coding of extracted qualitative data. Due to the low number of qualitative studies, we utilised reported themes, reported participant quotes and researcher analysis to maximise the amount of data available (Finfgeld‐Connett, 2018), and to allow for concepts to be ‘translated’ from one study to another; (2) establishing descriptive themes and developing new codes and (3) creating a final set of analytical themes. Across all three stages, the constructed themes were discussed between JM and SA and research team members, including those with personal experience of psychosis spectrum conditions. The qualitative literature was meta‐synthesised to generate a model of body image in people who experience psychosis to serve as a hypothesis‐generating framework to guide future clinical research, and Figure 2 was produced to describe the results.

#### Quantitative analysis

From the extracted data, it was clear there were not enough homogeneous data to conduct a formal meta‐analysis. Therefore, we followed the Synthesis Without Meta‐analysis (SWIM) guidelines (Campbell et al., 2020). We considered statistically significant relationships between measured variables. We included relationships between body image and psychosis or psychosis‐like experiences in studies including people with psychosis and the general population, or relationships between measured variables and body image in people diagnosed with psychosis spectrum conditions.

Body image is a heterogeneous construct, and we anticipated a range of measurements would be used. To aid the clarity of reporting, we have defined negative body image to mean negative evaluation of one's body (Cash et al., 2004). When other assessments of body image have been used, we have specified this within the reporting of results.

### Reflexivity

This review draws on a variety of secondary sources and was conducted by a team with a range of academic, clinical and personal experiences of body image concerns in psychotic spectrum conditions, who were drawn to conduct this review because they observed body image is an important issue in psychosis. Therefore, we present a short reflexivity section on what the team may have contributed to the analysis. Critical realist philosophy acknowledges people's experiences of body image as ultimately real for them, but socially shaped by culture, identity and other individual experiences (Waring & Kelly, 2023). Similarly, we wish to acknowledge that our interpretations of data in conducting this review and producing the report are likely shaped by our own backgrounds.

## RESULTS

### Study characteristics and study quality

There were 31 studies meeting criteria: 24 reported cross‐sectional quantitative findings, one reported longitudinal cross‐sectional research, five reported cross‐sectional qualitative research and one reported cross‐sectional mixed‐methods research. The overall mean quality rating was 3.26 (*SD* = 1.06) out of five—see Supplementary Appendix B for full scores. Initial study quality agreement by independent raters' (Cohen's kappa statistic) was .79.

The quantitative studies on average scored 2.96 (*SD* = 0.9) on quality criteria. While most studies utilised appropriate statistics and sampling approaches that were relevant to addressing the research question, they were limited by it being difficult to determine if the sample was representative of the target population. The qualitative studies scored 4.8 (*SD* = 0.44) on the quality criteria. Most studies used appropriate methodology to address the research question and reported qualitative data in detail to demonstrate coherence between data, collection, analysis and researcher interpretation. The single mixed‐methods study and single prospective study both scored 3.

In total 24 studies explored body image in people diagnosed with psychotic conditions, and 7 studies explored body image concerns and psychosis‐like experiences in the general population. The results come from 20,565 participants, of which 2127 were people experiencing psychotic conditions, 18,294 people from the general population (including 15,628 from epidemiological studies and 624 controls), 129 people with other conditions being compared to psychosis (such as bipolar without psychotic features) and 15 carers of people with psychotic conditions. The studies were conducted in the UK (*n* = 5), Australia (*n* = 5), India (*n* = 3), the USA (*n* = 3), Belgium (*n* = 2) and Poland (*n* = 2), with the remaining nine being one from each of China, New Zealand, Germany, Lebanon, Japan, Turkey, South Korea, Tunisia and Spain. In psychosis samples (*k* = 24), ethnicity or race was not reported in the demographics of 16 (66%) manuscripts, with the available weighted mean being 78.5% white. Of these psychosis samples (*k* = 24), nine were of inpatients, 13 outpatients and two mixed inpatient and outpatient samples. In studies reporting age (*k* = 20), the weighted mean participant age was 39.5, and for studies reporting gender (*k* = 23), 47.1% were female. The weighted mean BMI across studies reporting (*k* = 9) was 26.98, which falls in the overweight category. Further details about the studies can be seen in Tables 1, 2 and 3. Summary statistics are reported for control samples and ‘general’ populations in Supplementary Appendix C.

**TABLE 1:** Psychosis diagnosis.

Authors, reference, country, year and design	Sample characteristics	Diagnostic criteria	Body image measure	Related factors qualitative information	Associations with body image (*r*, unless otherwise stated)	Quality
Al Halabi, 2012, Spain Cross‐sectional survey	118 people diagnosed with schizophrenia, 41.23 years (*SD* unclear) for schizophrenia. Unclear how many were female Ethnicity was unclear Outpatients 93 people diagnosed with bipolar—following a check with the corresponding author, it was unclear how many people in the bipolar group had experienced psychosis	ICD‐10	BWISE, VAS‐BWI	1. CGI‐S (Guy, 1976)	BWISE −0.25, *p* < .001	3*
De Hert, 2006, Belgium Cross‐sectional survey	*N* = 300 34% female 36.8 years (*SD* = 11.9) years BMI: 26.3 (*SD* = 4.9), 99.3% were on antipsychotics 99% were described as Caucasian Belgian, illness duration unclear All were outpatients	DSM‐IV diagnosis of schizophrenia or schizoaffective disorder	BWISE	1. BMI	BMI = −.30	3*
				1. Presence of metabolic syndrome	Compared to patients with normal glucose values and patients with prediabetic abnormalities had significantly lower body image.	
				1. Different antipsychotics	No significant differences in body image scores between antipsychotic types (olanzapine, risperidone, clozapine, quetiapine, amisulpride and aripiprazole)	
Dikmen, 2022, Turkey Cross‐sectional survey	*N* = 154 41.6% female 43.40 years (*SD* = 10.59) Outpatients Ethnicity: unclear	DSM‐5	BWISE	1. Medication adherence—MARS (Thompson et al., 2000)	Psychosocial adjustment subscale = .312 (*p* = <.05)	3*
Every‐Palmer, 2018, New Zealand Cross‐sectional Mixed methods	*N* = 51 22% female 38 (*SD* = 10.4) years BMI: 35.3 (*SD* = 8.1) Ethnicity was Maori (65%), White (29%), Pacific islander (26%), other 6% Inpatients	ICD‐10 diagnosis of psychosis 78% schizophrenia, 6 (12%) schizoaffective, 2 (4%) bipolar with psychotic features and 3 (6%) unspecified psychosis	Assessed via interview and bespoke Likert scale item on body satisfaction	2. Having a larger body was positively appraised for providing protection. 2. Over 60% reported ‘being unhappy with their appearance’.	N/A	3*
Goyal, 2019, India Cross‐sectional survey	*N* = 203 54.6% female 38.21 (*SD* = 10.32) years Ethnicity: unclear BMI: 28.04 (*SD* = 5.21) All were outpatients	DSM‐IV‐TR criteria for schizophrenia—confirmed with MINI	BWISE	Screening question ‘Have you gained some weight on your present medications?’ with yes/no response.	Patients reporting recent weight gain had lower BWISE scores	2*
Hassamal, 2017, USA Cross‐sectional survey	*N* = 80 40% female 40.0 (*SD* = 11.88) years BMI: 26.87 (*SD* = 5.90) Ethnicity: 31 White (38.75%) 49 African American (61.25%) Inpatients 36 controls with non‐psychotic mood disorders, Inpatients	Diagnosed with psychotic conditions according to DSM‐IV‐TR criteria	SFRS—discrepancy (Stunkard & Sorensen, 1983)	Attitudes towards obesity OAS (Allison et al., 1991)	Ideal body‐self was not significantly different in psychotic disorders vs. non‐psychotic disorders. There were no differences in attitudes towards obesity.	2*
Lee, 2021, South Korea Cross‐sectional survey	*N* = 147 100% female 34.65 (*SD* = 8.20) years BMI: 25.23 Outpatients Ethnicity: unclear	Diagnosed with a psychotic condition (schizophrenia or bipolar) by a psychiatrist	BIS (Kim & Park, 1995)	1. Medication adherence MARS (Korean version)	= −0.616, *p* < .001	3*
				1. Insight—SUMD (Korean version) (Amador et al., 1991)	−0.397, *p* < .001	
				1. Mental health confidence—MHCS (Korean version) (Carpinello et al., 2000)	0.565, *p* < .001	
Lundgren, 2014, USA Cross‐sectional survey	*N* = 22 55.4% female 36.5 (*SD* = 7.5) years BMI 36.5 (*SD* = 7.5) Race demographics: 63.6% African American 18.2% Latinx 13.6% white—4.5% unclear Outpatients *N* = 27 controls	All diagnosed with schizophrenia spectrum conditions according to DSM‐IV	Weight and Lifestyle Inventory (Wadden & Foster, 2006)	2. Compared body image to controls who met criteria for obesity.	Less satisfied with current shape and weight, but not less satisfied with appearance overall.	3*
Oh, South Korea, 2017 Cross‐sectional survey	*N* = 167 47.3%, female Age ranges: 20–30 (15.0%) 31–50 (28.1%) 51–60 (41.3%) Inpatients Ethnicity: unclear	All diagnosed with schizophrenia according to DSM‐IV‐TR criteria	MBSRQ (Cash, 2000)	1. Self‐Esteem RES (Rosenberg, 1989)	Body appearance orientation = 0.52, *p* < .001 Body appearance satisfaction = 0.34, *p* < .001	5*
				1. Gender	Females reported higher body appearance orientation than males *d* = 0.40, (*p* = .017) but not satisfaction (n.s)	
				1. BMI	n.s (Body appearance focus or Body Satisfaction)	
				1. Education Level	n.s (Body appearance focus or Body Satisfaction)	
				1. Smoking status	Smokers has lower body appearance orientation *d* = 0.33, *p* = .026, Body satisfaction n.s	
Pindikura, 2022, India Cross‐sectional survey	*N* = 50 41.3% female Age unclear Ethnicity: unclear Outpatients	All patients diagnosed with schizophrenia (ICD‐10)	BWISE	1. BMI	= −0.359, *p* = .023	3*
				1. Self‐Esteem RES (Rosenberg, 1989)	= 0.397, *p* = .01	
				2. Compared to controls	n.s difference in body image	
Tham, 2009, Australia Cross‐sectional survey	*N* = 42 23.8% female Mean age 40 Ethnicity: unclear Outpatients	All diagnosed with psychotic condition but diagnostic system unclear	A Body figure rating scale developed for Caucasians (Bulik et al, 2001)	2. Difference in perceived, preferred and actual body image.	*t*‐tests revealed a significant difference between the perceived, preferred and actual figures.	2*
				2. Gender	*t*‐tests revealed no significant difference between perceived body size in female participants.	
Vancampfort, 2011, Belgium Cross‐sectional	*N* = 60 36.6% female 38.1 years (*SD* = 26.7) Inpatients BMI: 26.7 Ethnicity: Unclear *N* = 40 controls	Diagnosed with schizophrenia according to DSM‐IV	Body attractiveness: The physical self‐perception profile (Fox & Corbin 1989	1. Six minute walk distance	= −0.40, *p* = <.001	4*
				1. BMI	= 0.20 n.s	
				1. Recall of physical activity levels in past 12 months (Baecke et al., 1982)	Sports participation = 0.40, *p* < .05	
				2. Compared to controls	People diagnosed with schizophrenia reported lower body attractiveness 12.2 (*SD* =4.4) *p* < .001	
				2. Compared by weight class	People diagnosed with schizophrenia who were obese rated lower body attractiveness 9.2 (*SD* = 3.9) than overweight people 12.7 (*SD* = 3.9), *p* < .001	
Wong, 2009, Hong KongCross‐sectional survey	*N* = 87 50.6% female 18.92 (*SD* = 1.46) years. Controls = 102	ICD‐10 criteria for schizophrenia spectrum condition	Body image questionnaire comprising two parts—the FRS (Collins, 1991) and cognitive attitude towards body size (Shih & Kubo, 2002)—was used to assess body and weight satisfaction	2. Association between current figure and ideal figure	= 0.24 (*p* < .05).	2*
				2. Gender	Noted to be differences between males and females in body image but analysis unclear.	

Abbreviations: BIS, Body Image Scale; BWISE, Body Weight, Image and Self‐Esteem Evaluation Questionnaire; CGI‐S, Clinical Global Impressions Scale—Severity; FRS, Figure Rating Scale; IAS, Image of Ageing Scale; MARS, Medication Adherence Scale; MBSRQ, Multidimensional Body‐Self Relations Questionnaire; MHCS, Mental Health Confidence Scale; OAS, Obesity Attitudes Scale; SAUMD, Scale to Assess Unawareness of Mental Disorder; SFRS, Stunkard Figure Rating Scale; VAS‐BWI, Visual Analogue Scale for Weight and Body Image.

**TABLE 2:** Psychosis symptoms.

Reference, country, year and design	Sample characteristics	Diagnostic criteria	Body image measure/s	Psychosis	Associations with body image (*r*, unless otherwise stated)	Quality
		Psychosis assessment		Other correlates considered		
Bagrowska, 2022, Poland Cross‐sectional survey	*N* = 539 65.5% female 33.44 years (*SD* = 12.03) years BMI: 24.62 (*SD* = 5.96) Ethnicity: unclear	1. General population study (allowed self‐reporting of diagnosis) 2. R‐GPTS‐Polish version (Freeman et al., 2021)	KWCO, BES (Franzoi & Shields, 1984), BDS (Mutale et al., 2016)	2. Self‐esteem.RES (Rosenberg, 1989)	Indirect path from body image to paranoia via self‐esteem: (*β* = −0.07, 95% CI = −0.112 to −0.031)	3*
				1. Paranoia. R‐GPTS	BES = −0.403 (*p* < .001) Body dissatisfaction and paranoia 0.276, *p* < .001	
				2. Affect PANAS (Watson et al., 1988)	Indirect path from body image to paranoia via negative emotions *β* = −0.128, 95% CI = −0.171 to −0.087	
				2. BMI	Persecutory paranoia (0.08, *p* < .05)	
				2. Rejection sensitivity RSS (Downey & Feldman, 1996)	Indirect path from body image to paranoia via rejection sensitivity *β* = −0.027, 95% CI = −0.051 to −0.008	
Fekih‐Romdhane, 2023, Tunisia Prospective study	*N* = 510 61.2% female 16.05 years (*SD* = 1.01) Ethnicity: unclear	1. General population 2. CAPE‐42 (Konings et al., 2006)	MBSRQ (Brown et al., 1990)	1. PLEs CAPE (Konings et al., 2006)	Baseline PLEs influenced Body area Satisfaction (X → M1 = −0.1133, *p* < .001), which influenced subsequent Body Area Satisfaction at 6 and 12 months (M1 → M2 = 0.6392, *p* < .001 and M1 → M3 = 0.1729, *p* < .001, respectively).	3*
Keating, 2016, Australia Cross‐sectional survey	*N* = 226 71.6%, female 25.4 years (*SD* = 6.05) Ethnicity: unclear	1. General population 2. PDI (Peters et al., 2004) 2. ASI (Cicero et al., 2010)	DCQ (Oosthuizen et al., 1998)	1. Delusions PDI (Peters et al., 2004)	= 0.56. *p* = .01. Predicted body image concern.	3*
				1. Aberrant Salience ASI (Cicero et al., 2010)	= 0.44, *p* = .44 Did not predict body image concern.	
Koide, 2002, Japan Cross‐sectional survey	*N* = 93 52.6% female 48.2 years (no *SD*) Ethnicity: unclear Inpatients Controls = 177	1. Diagnosed with schizophrenia according to DSM‐IV criteria 2. SAPs and SANS	BIQ (Koide, 1985)	1. Positive Symptoms SAPS (Andreasen, 1984) 1. Negative Symptoms SANS (Andreasen, 1990)	High scores on positive and negative symptoms associated with high powerlessness.	2*
Lavell, 2014, AustraliaCross‐sectional survey	*N* = 246 74% female 21.13 years (*SD* =44.86)	1. General population 2. PDI	AAI (Veale et al., 2014)	1. Delusions PDI (Peters et al., 2004)	= 0.41, *p* < .01 Explained 1.4% of variance in hierarchical regression considering gender, age, obsessive compulsive thinking and social anxiety (*p* = .017)	3*
Mahfoud, 2023, Lebanon Cross‐sectional survey	*N* = 204 42.6% female 55.57 years (*SD* = 11.30 years) 37.25% were overweight (BMI >25) Inpatients	1. All diagnosed with schizophrenia according to DSM‐5 2. PANSS (Kay et al., 1987)	BAS‐2 (Tylka & Wood‐Barcalow, 2015a) FAS (Alleva et al., 2017)—Arabic Version	1. Psychosis symptoms PANSS	FAS = − 0.26 (*p* < .05)	5*
				2. Age	FAS = 0.31 (*p* < .001)	
				2. BMI	BAS = 0.29 (*p* = <.05)	
				2. Weight self‐stigma WSSQ (Lillis et al., 2010)	*D* = 0.289, *p* = .048 (comparing obese weight class to non‐obese weight class)	
Malcolm, 2022, Australia Cross‐sectional survey	*N* = 114 100% female 31.37 (*SD* = 11.17) years BMI: 25.84 (*SD* = 7.01)	1. General population sample 2. TPS (Fenigstein & Vanable, 1992)	SFRS (Stunkard & Sorensen, 1983).	1. Paranoia TPS (Fenigstein & Vanable, 1992)	Shape concern had significant positive relationship with paranoia (=0.49, *p* < .01) and weight concern (=0.42, *p* = <.01). Exploratory mediation analysis suggested paranoia‐mediated relationships between feeling larger and having appearance concerns (*B* = −.25, total effect confidence interval did not include zero).	2*
Röhricht, 2002, Germany Cross‐sectional survey	*N* = 60, 60% female 35.9 years (*SD* 11.1) Unclear ethnicity Inpatients	1. All diagnosed with schizophrenia according to DSM‐IV 2. Levels of somatic hallucinations were developed using a cluster analysis based on results from BSABS (Gross et al., 1987)	‘How satisfied are you with your body?’ 0–10	1. Somatic hallucination levels	Cluster analysis of three patient groups with differing levels of ‘somatic hallucination’—n.s difference in body satisfaction	2*
Sakson‐Obada, Poland, 2018 Cross‐sectional survey	N = 63 41.3% female 29.5 years (*SD* = 5.5) BMI: 26.8 (6.7) *N* = 63 controls Inpatients	1. Patients diagnosed with schizophrenia spectrum disorders according to ICD‐10 criteria 2. PANSS (Kay et al., 1987)	BSQ (Sakson‐Obada & Wycisk, 2015)	1. PANSS	Satisfaction with appearance, positive PANSS =0.28 (*p* < .05), All other PANSS n.s Satisfaction with fitness, all PANSS scores n.s	2*
				2. BMI	n.s (Satisfaction with Appearance or Fitness)	
				2. Age	n.s (Satisfaction with Appearance or Fitness)	
				2. Gender	Difference in satisfaction with appearance (*M* female = 2.8, *M* male =1.9; *t* = 4.3, *p* = .001)	
				2. Years of education	n.s (Satisfaction with Appearance or Fitness)	
				2. Age of onset	n.s (Satisfaction with Appearance or Fitness)	
				2. Duration of illness	n.s (Satisfaction with Appearance or Fitness)	
				2. Relapse number	n.s (Satisfaction with Appearance or Fitness)	
				2. Time since last episode	n.s (Satisfaction with Appearance or Fitness)	
				2. Number of hospitalisations (psychosis)	n.s (Satisfaction with Appearance or Fitness)	
				2. Number of nonpsychiatric hospitalisations	n.s (Satisfaction with Appearance or Fitness)	
Toh, Australia, 2023 Cross‐sectional survey	*N* = 407 44% female, 29.1 years Ethnicity unclear	1. Self‐reported diagnosis (no psychosis) 2. The perceptual aberration and magical ideation measure Chapman (1978) and Eckbald and Chapman (1983) 2. TPS Fenigstein and Vanable (1992), 2. PIQ McKay, Langdon and Coltheart (2006)	Body Dysphoria—DCQ (Mancuso, Knoesen and Castle 2010) Body Consciousness—BCQ (Miller, Murphy & Buss 1981)	1. PIQ Persecutory ideation	DCQ = 0.362, *p* < .01	3*
				1. Perceptual aberration and magical ideation measure—Magical Thinking	DCQ = 0.23, *p* < .01 BCQ (public) = 0.17, *p* < .01	
				1. TPS Paranoia	DCQ = 0.48, *p* < .01 BCQ (public), 0.15, *p* < .01 In regression models, paranoia, but not persecutory ideation, significantly contributed to the prediction of dysmorphic concerns, alongside sex and negative emotional states. In regression models, paranoia did not significantly contribute to the prediction of public or private body consciousness, nor body competence.	
Waite, 2017, USA Secondary analysis of cross‐sectional survey (NCS‐R) and NCS‐A	*N* = 15,628 NCS‐R sample (*n* = 5515), 44.73 years (*SD* = 17.5), 52.6% female NCS‐A (*n* = 10,113), 15.18 (*SD* = 1.50), 52.6% female in NCS‐R sample only—60% (weighted) of participants had a BMI categorised as overweight (33.6%) or obese (26.4%).	1. General population 2. Two paranoia measures, one measuring mild paranoia and the other severe.	Single item asking if people have ever experienced worries about being overweight.	Concerns about weight were associated with paranoia in the NCS‐R (OR = 1.48, *p* = .006, CI = 1.123, 1.955) and NCS‐A (OR = 1.67, *p* < .001, CI = 1.490, 1.873). The associations remained significant after controlling for gender and body mass index.		4*
Waite, 2019, UK Cross‐sectional survey	*N* = 60, 40% female, 41.9 years (*SD* = 11.7) Mixed inpatient/outpatient 81.6% white, 6.6% Black Caribbean, 5% Indian, 3.3% Pakistani, 1.6% Black African, 1.6% Other	1. All diagnosed with psychosis spectrum and experiencing persecutory paranoia. 2. VOCA (Waite et al., 2019) 2. CAPS (Bell et al., 2006) 2. GPTS^38^ (Green et al., 2008)	BESSA	1. Voice Hearing VOCA	91.7% reported that voices have made comments about their appearance and 88.3% reported hearing voices commenting on their appearance on at least a weekly basis. 90% was negative and 65% positive.	3*
				1. Anomalous Perceptions CAPS	Negative voice content = 0.47, *p* < .001 Positive Voice Content = −0.033, n.s	
				1. Paranoia GPTS	Negative voice content = 0.37, *p* < .01 Positive voice content = 0.028, n.s	
				2. BMI	BMI was significantly positively associated with specific voice comments, for example ‘people think I am fat’ (*r* = .421, *p* = .001) and negatively correlated with ‘I am too skinny’ (*r* = −.354, *p* = .007).	
				2. Depression BDI	Negative voice content = 0.326, *p* < .05 Positive voice content = − 0.48, *p* < .05	
				2. Self‐Schemas BCSS—Negative Self‐Beliefs	Negative voices = 0.47, *p* < .01 Positive voices, = −0.38, *p* < .02	
				2. Self‐Schemas BCSS—Positive Self‐Beliefs	Negative voices = −0.32, *p* < .05 Positive voices = 0.26, *p* < .05	
				2. Insomnia ISI (Bastien et al., 2001)	Negative voice content = −0.44, *p* < .01 Positive Voice Content = 0.056, n.s	
				2. Safety Behaviours SBQ	Negative voice content =0.366, *p* < .01 Positive Voice Content =	
				2. Wellbeing WEMWBS (Tennant et al., 2007)	Negative voice content Positive voice content =0.456., *p* < .001	
				2. Time Budget (Jolley et al., 2006)	n.s	
				2. Worry PSWQ (Meyer et al., 1990)	Negative voices = 0.27, *p* < .05 Positive voice content	
Waite, Diamond, et al., 20222, UK Cross‐sectional survey with comparison to controls	*N* = 115 40% female 41.8 years (*SD* = 11.8) Inpatients and outpatients Ethnicity reported—85.2% white *N* = 200 controls	1. All diagnosed with psychosis spectrum and experiencing persecutory paranoia. R‐GPTS (Freeman et al., 2021) 2. CAPS (Bell et al., 2006) 2. TEPS (Bell et al., 2006)	BESAA (Mendelson et al., 2001)	1. Paranoia R‐GPTS (Freeman et al., 2021)	Ideas of reference = −0.24, *p* < .05 Persecution = −0.25, *p* < .05	4*
				1. Anomalous Perceptions CAPS (Bell et al., 2006)	= −0.21, *p* < .05	
				1. Anhedonia TEPS (Bell et al., 2006)	Anhedonia = −0.33, *p* < .01	
				2. BCSS (Fowler et al., 2006)	Negative self‐belief −0.51, *p* < .01 Positive self‐belief n.s	
				2. Depression BDI‐II (Beck et al., 1996)	= −0.55, *p* < .01	
				2. Suicidality CSSRS (Posner et al., 2011)	= −0.321, *p* < .01	
				2. Worry PSWQ (Meyer et al., 1990)	−0.437, *p* < .01	
				2. Insomnia ISI (Bastien et al., 2001)	−0.300, *p* < .01	
				2. Safety Behaviours SBQ (Freeman et al., 2001)	−0.384, *p* < .01	
				2. SCOFF item—binge eating (Morgan et al., 1999)	Fifty‐eight (51.8%) patients reported a loss of control over eating. There were significant differences in body esteem [*t*(110) = 4.30, *p* < .001] between those reporting a loss of control over eating (M = 1.06, *SD* = 0.66) and those who did not (*M* = 1.59, *SD* = 0.64).	
				2. Wellbeing WEMWBS (Tennant et al., 2007)	= 0.41. *p* < .01	
				2. Quality of Life EQ‐5D‐5 (Herdman et al., 2011)	Quality of life = 0.22, *p* < .01 Overall health = 0.31, *p* < .01	
				2. Time Budget (Jolley et al., 2006)	n.s	
				2. Gender	Significant differences between male (*M* = 34.25, *SD* = 15.73) and female (*M* = 24.08, *SD* = 15.11) participants in total body esteem [*t*(113) = 3.45, *p* = .001] and for the appearance [*t*(110) = 3.37, *p* = .001] and weight [*t*(110) = 4.04, *p* < .001] subscales, but not the attribution subscale [*t*(105) = 0.45, *p* = .66].	
				2. BMI	Levels of body esteem were statistically significantly lower in the overweight (1.29 ± 0.69, *p* = .022) and obese (1.13 ± 0.64, *p* < .001) BMI groups than the normal weight (1.74 ± 0.65) category.	

Abbreviations: AAI: Appearance Anxiety Inventory, ASI, Aberrant Salience Inventory; BAS, Body Appreciation Scale‐2; BCSS, Brief Core Schemas Scale; BCQ, Body Consciousness Questionnaire; BES, Body Esteem Scale; BDS, Body Dissatisfaction Scale; BIQ, Body Image Questionnaire; BMI, Body Mass Index; BSABS, Bonn Scale for the Assessment of Basic Symptoms; BSQ, Bodily Self‐Questionnaire; CAPE, Community Assessment of Psychotic Symptoms; CAPS, Cardiff Anomalous Perceptions Scale; CSSRS, Columbia‐Suicide Severity Rating Scale; DCQ, Dysphoric Concerns Questionnaire; EQ‐5D‐5, 5‐level EQ‐5D version; FAS, Functionality Appreciation Scale; GPTS, Green et al. Paranoid Thoughts Scale; KWCO, the body image questionnaire according to Głębocka; PDI, Peters' Delusions Inventory; PSWQ, Penn State Worry Questionnaire; R‐GPTS, Revised Green et al. Paranoid Thoughts Scale; PANSS, Positive and Negative Syndrome Scale; PANAS, Positive and Negative Affect Schedule; PLEs, Psychotic‐Like Experiences; PIQ, Persecutory Ideation Questionnaire; RES, Rosenberg Self‐Esteem Scale; RSS, Rejection Sensitivity Scale; SAPS, Scale for the Assessment of Positive Symptoms; SANS, Scale for the Assessment of Negative Symptoms; SBQ, Safety Behaviours Questionnaire; TPS, The Paranoia Scale; VOCA, Voices Commenting on Appearance Scale; WEMBS, Warwick Edinburgh Wellbeing Scale; WSSQ, Weight self‐stigma questionnaire.

**TABLE 3:** Qualitative papers.

Reference, country, year and design	Sample characteristics	Diagnostic criteria	How body image assessed	Key themes	Quality
Amatullah, 2020, India Cross‐sectional qualitative Interview	25‐year‐old male with drug induced psychosis. Inpatient	DSM‐5 criteria used during psychiatric hospital admission.	Interview	Internationalisation of cultural appearance norms. Psychotic disorders can violate cultural appearance norms.	5*
Becker, 2022, Chile Cross‐sectional qualitative	*N* = 18 55.6% female 46 (*SD* = 13.76) years Ethnicity not reported Outpatients	Diagnostic criteria unclear—states diagnosis made by psychiatrist.	Interview	Chilean cultural values foreground importance of looking neat and decent. Psychotic disorders can violate cultural appearance norms	4*
Marshall, 2020, UK Cross‐sectional interview	*N* = 12 50% female 26.3 (*SD* = 10.6) years 91% white, 9% Chinese Outpatients	All diagnosed with a schizophrenia spectrum condition	Interview	Ambivalence towards body image, body image impacted by uncontrollable medication side effects, negative self‐concept, appearance as a source of threat, emotional impact of poor body image, double stigma of having psychosis and non‐normative appearance.	5*
Waite, 2022, UK Cross‐sectional interview	N = 10 30% female 36.6 years (*SD* = 15.2) Outpatients	All diagnosed with a schizophrenia spectrum condition	Interview	Ambivalence towards body image, previous appearance‐based bullying and abuse may provide expectations of treatment by others.	5*
White, 2021, UK Cross‐sectional interview	N = 10 Age: 29.7 for men, 50.0 for women 40% female Outpatients	All were diagnosed with a schizophrenia spectrum condition	Interview	Double stigma of having psychosis and non‐normative appearance, feeling unattractive.	5*

### Quantitative studies

#### Body image measurements

Body image was indexed in psychosis populations using a range of measures, including three bespoke single‐item measures. The most frequently used assessment scale of body image in people diagnosed with psychotic conditions was the Body Weight and Self‐Esteem (BWISE) (*k* = 5), which usually has three subscales. Tables 1 and 2 report measures used across studies. Table 4 reports more information about the body image scales.

**TABLE 4:** Scales used to assess body image in psychotic conditions.

Measurement used for body image concerns	Frequency of usage	Measures	Items	Definition of body image	General evidence of reliability/validity in psychosis
BIQ (Koide, 1985)	1 (Koide, 1985)	Assesses anatomical, functional and psychological aspects of body image	32 (reduced from 59 items with factor analysis in included study)	Higher scores indicate higher endorsement	NR
BAS‐2 (Tylka & Wood‐Barcalow, 2015a)	1 (Mahfoud et al., 2023)	Assesses positive body image as a single construct	10	Positive body image—higher scores indicate better positive body image	NR
Body figure rating scale developed for Caucasians^47^	1 (Tham et al., 2007)	Nine pictographs of Caucasian bodies are presented. Participants are invited to chose ‘preference, perceived and actual body’ shape.	9	Differences between preference, perceived and actual taken to indicate body image	NR
BIS (Kim & Park, 1995)	1 (Lee & Jang, 2021)	Two domains, physical appearance and physical health	23	High scores indicate poor body image	Internal reliability 0.87
BESAA (Mendelson et al., 2001)	2 (Waite et al., 2019; Waite, Diamond, et al., 2023	Three subscales: appearance (general feelings towards appearance), attributions (evaluations of appearance) and weight (satisfaction about weight).	23	Higher scores indicate higher body esteem	Excellent reliability (internal consistency) observed in a mixed general/psychotic conditions sample (Waite, Diamond, et al., 2022)
BSQ (Sakson‐Obada & Wycisk, 2015)	1 (Sakson‐Obada et al., 2018)	Disturbances in body functions (perception, interpretation, regulation), sense of physical identity, and three aspects of body image (appearance evaluation, fitness evaluation, acceptance of biological sex)	78 (women), 81 (men)	Higher scores indicate disturbances in area assessed	NR
BWISE (Awad & Voruganti, 2004)	5 (Al‐Halabi et al., 2012; De Hert et al., 2006; Goyal et al., 2019; Pindikura et al., 2023; Türkoğlu Dikmen et al., 2022)	Factor analysis (De Hert et al., 2006) yielded three factors General distress related to body image General well‐being and activity Knowledge and behavioural intentions about weight gain.	12	Higher scores indicate better adjustment to body image issues	Different factor structures used (Al‐Halabi et al., 2012; De Hert et al., 2006; Goyal et al., 2019; Türkoğlu Dikmen et al., 2022)—internal reliability varied.
Cognitive attitudes towards body size	1 (Wong et al., 2009)	Participants were asked if they felt they were underweight, of normal weight, or overweight and whether they would like to gain weight, stay the same, or lose weight	1		NR
FAS (Alleva et al., 2017)	1 (Mahfoud et al., 2023)	Measures appreciation for body function	7	Higher scores indicate more appreciation for body functionality	NR
Figure Rating Scale (Collins, 1991)	1 (Wong et al., 2009)	Seven male and female bodies were presented—participants were invited to pick what body represented their current figure and their ideal figure	7	Dissatisfaction with body figure could be calculated by the difference between current and ideal figure	NR
MBSRQ (Cash, 2000)	1 (Oh et al., 2017)	Included study used two subscales: appearance orientation subscale (12 items) and the body areas satisfaction subscale (9 items)	21 relevant items	Higher scores indicate contentment with body image	Excellent internal reliability for two subscales 0.81 and 0.88
SFRS (Stunkard & Sorensen, 1983)	1 (Hassamal et al., 2017)	The scale consists of nine female and male figure drawings depicting very low adiposity (1) to very high adiposity (9). Participants were asked to select which figure they think they currently look like (‘think’ condition) and which figure represents how they currently feel in their body (‘feel’ condition).	1	A discrepancy indicates poor body image.	NR
Single item	1 (Röhricht & Priebe, 2002)	‘How satisfied are you with your body?’ 0–10	1	Higher scores indicate higher body satisfaction	NR
Single item	1 (Al‐Halabi et al., 2012)	Measures satisfaction with weight on a 100 mm visual analogue scale with ‘very satisfied’ and ‘very unsatisfied’ at either end.	1	Higher scores indicate more weight dissatisfaction.	NR
The physical self‐perception profile (Fox & Corbin 1989	51	Five subscales: perceived sport competence, perceived physical condition, perceived body attractiveness, perceived physical strength and perceived physical self‐worth	30	Higher scores indicate more positive perceptions	NR
Three Single‐Item Questions	1 (Every‐Palmer et al., 2018)	Are you worried about your body shape or your appearance? Do you think your body shape is worse than other people's: do you compare yourself negatively to others' body shape? Do you become self‐conscious and worried about your body shape when around other people?	1	Higher scores indicate worse body image.	NR
Weight and Lifestyle Inventory (Wadden & Foster, 2006)	1 (Lundgren et al., 2014)	Single items asking about satisfaction with weight, shape and appearance	3 relevant items	Higher indicate lower satisfaction	NR
Weight Self‐Stigma Questionnaire (Lillis et al., 2010)	(Mahfoud et al., 2023) 1	Self‐devaluation and fear of enacted stigma	12	Higher scores indicate more weight stigma	NR

#### Section 1—The relationship between psychosis symptoms and body image

One of the aims of this review was to summarise research examining the relationships between body image and psychosis symptoms. Fourteen of the thirteen studies exploring this were cross‐sectional and considered people from the general population and psychosis spectrum disorder samples. Six studies focused on paranoia; one of these also assessed anhedonia and hallucinatory experiences, while another included voice hearing. Two studies examined broader positive symptoms; two focused specifically on delusional‐like beliefs. One study investigated somatic hallucinations; another assessed both positive and negative symptoms together. Finally, one study explored psychosis‐like experiences in the general population.

##### Positive psychotic symptoms

In people diagnosed with schizophrenia, there was a small positive correlation between positive symptom severity and body image appearance dissatisfaction (*r* = .28, *p* < .05), and positive symptoms (*β* −0.92, *p* < .001). Female gender (*β* = −1.44, *p* < .001) predicted dissatisfaction with appearance (Sakson‐Obada et al., 2018). A further study noted a negative correlation between body functionality appreciation and positive symptoms (*r* = −.26, *p* < .05) in people diagnosed with schizophrenia (Mahfoud et al., 2023).

##### Paranoia

From the general population, a measure of paranoia was significantly associated with lower levels of body image (OR = 1.67) (Waite & Freeman, 2017), concerns with shape (*r* = .49, *p* < .01) and weight (*r* = .42, *p* < .01), lower reported levels of body esteem (*r* = −.40, *p* < .01) and higher body dissatisfaction (*r* = .27, *p* < .01) (Bagrowska et al., 2022). Moreover, paranoia‐mediated relationships between feeling larger and having appearance concerns in a study conducted in a non‐clinical sample (*B* = −.25, total effect confidence interval did not include zero) (Malcolm et al., 2022). In another non‐clinical sample study, paranoia, but not persecutory ideation, significantly contributed to the prediction of body dysmorphic symptoms (Toh et al., 2023).

In people with psychotic disorders in the UK, there was a negative relationship between body esteem and both ideas of reference (*r* = −.24, *p* < .05) and persecution (*r* = −.25, *p* < .05) (Waite, Diamond, et al., 2022). Moreover, there was a positive correlation between people who heard voices commenting negatively on their appearance and paranoia (*r* = .37, *p* < .01) (Waite et al., 2019) in people diagnosed with psychosis.

##### Delusion proneness

Two Australian studies conducted in the general population suggested that an assessment of ‘delusional proneness’ had a large positive correlation with body image concerns (*r* = .56, *p* < .001) (Keating et al., 2016) and body dysmorphia symptoms (*r* = .41, *p* < .01) (Lavell et al., 2014).

##### Voice hearing

One UK study (Waite et al., 2019) examined voice hearing experiences in people diagnosed with schizophrenia and found that over 91% of people reported that voices commented on their appearance. Hearing voices that commented positively (65% frequency) on the body had large to medium positive correlations with body image esteem appearance (*r* = .49, *p* < .001), attribution (*r* = .58, *p* < .001) and esteem (*r* = .42, *p* = .001) subscales. Whereas, hearing negative comments (90% frequency) about their body demonstrated negative correlations with the body image esteem appearance (*r* = −.45, *p* < .001), attribution (*r* = −.30, *p* < .001) and esteem (*r* = −.37, *p* = .004) subscales.

##### Somatic hallucinations

A cluster analysis study indicated that patients diagnosed with schizophrenia reporting differing levels of somatic hallucinations did not differ in their body satisfaction (Röhricht & Priebe, 2002).

##### Hallucinations

In people diagnosed with psychosis experiencing frequent persecutory delusions, there was a negative relationship between hallucinatory experiences and body esteem (*r* = −.21, *p* = .025) (Waite, Diamond, et al., 2022).

###### Negative symptoms

The previous study also showed a significant negative correlation between anhedonia and body image (*r* = −.33, *p* < .001) in people with psychotic conditions who experience frequent persecutory delusions (Waite, Diamond, et al., 2022).

###### Studies reporting a composite of positive and negative symptoms

In one study, people diagnosed with schizophrenia who present with higher levels of both positive and negative symptoms may perceive their body as being ‘powerless’ compared to those with lower symptoms (Koide et al., 2002).

###### Psychotic‐like experiences

A single study conducted in Tunisia examined longitudinal relationships between body image and psychotic‐like experiences in teenagers using a mediation model. It found that baseline psychotic‐like experiences (PLEs) influenced lower body area satisfaction at baseline (PLEs → body area satisfaction = −0.1133, *p* < .001), which influenced subsequent body area satisfaction at 6 and 12 months (baseline body area satisfaction → body area satisfaction 6 months = 0.6392, *p* < .001, and body area satisfaction 6 months → body area satisfaction 12 months = 0.1729, *p* < .001, respectively) (Fekih‐Romdhane et al., 2023).

###### General psychopathology

In people diagnosed with schizophrenia in Spain, higher Clinical Global Impression (CGI) scores were negatively associated with lowered body image, with a small effect size (*r* = −.25, *p* < .001) (Al‐Halabi et al., 2012).

#### Section 2—Other correlates

Psychological and behavioural correlates were extracted and described from 16 studies conducted with psychosis populations only (this included 3 studies that reported correlates in addition to relationships between body image and psychosis symptoms that are described in the previous section). We divided these into five main types, which were cognitive factors, emotional factors, behavioural factors, and health and social functioning.

##### Cognitive factors

###### Self‐esteem and self‐schema

In a regression model considering the effects of smoking, gender, education and past employment, body image satisfaction explained 33.6% of variance on self‐esteem in people diagnosed with schizophrenia (Oh et al., 2017). A further Indian study showed a positive correlation between body image and self‐esteem (Pindikura et al., 2023), and another study conducted in South Korea showed self‐esteem had a large positive correlation with body appearance orientation (*r* = .52, *p* < .001) and a small correlation with body image satisfaction (*r* = .34, *p* < .001) (Oh et al., 2017) suggesting that it might be protective in people diagnosed with psychotic conditions.

One UK quantitative study demonstrated a large correlation between negative self‐schemas and body image (*r* = −.516, *p* < .001) in people diagnosed with schizophrenia (Waite, Diamond, et al., 2022). In people who hear frequent voices, those who heard voices in the past week had a positive correlation between negative self‐schemas and negative voice content (*r* = .47, *p* < .001). For positive voice content, there was a positive correlation with positive self‐schemas (*r* = .26, *p* < .043) (Waite et al., 2019).

##### Emotional factors

###### Low mood and suicidal ideation

Evidence from people experiencing recent persecutory delusions showed body esteem had a large negative correlation with depression (*r* = −.55, *p* < .001) and a smaller correlation with suicidal ideation (*r* = −.32, *p* < .001) (Waite, Diamond, et al., 2022).

##### Behavioural factors

###### Medication adherence

Two studies explored relationships between body image and medication adherence and suggested that higher medication adherence is cross‐sectionally associated with better body image in psychosis. One study conducted in Turkey explored antipsychotic medication adherence in people diagnosed with schizophrenia and reported a significant positive relationship between self‐reported medication adherence and body image (*r* = .31, *p* < .05) (Türkoğlu Dikmen et al., 2022). A further study in women living with psychotic conditions including schizophrenia and bipolar disorder in South Korea showed a negative correlation between body image and medication adherence (*r* = −.616, *p* < .001) using a scale where higher scores indicated poorer body image (Lee & Jang, 2021).

###### Eating behaviour

Fifty‐eight (51.8%) patients with persecutory delusions reported a loss of control of eating. There was a significant reduction in body esteem [*t*(110) = 4.30, *p* < .001] between those reporting a loss of control over eating (*M* = 1.06, *SD* = 0.66) compared to those who did not (*M* = 1.59, *SD* = 0.64) (Waite, Diamond, et al., 2022). Moreover, 43% of participants diagnosed with psychotic conditions in a long‐stay forensic ward in New Zealand reported eating when not hungry to cope with negative emotions (Every‐Palmer et al., 2018).

##### Health and social functioning

###### Weight

Evidence for a relationship between BMI and body image in psychosis was mixed and may depend on the assessment method used. A negative correlation between body image and BMI (*r* = −.30, *p* < .01) (De Hert et al., 2006) was reported in Belgium, and India (*r* = −.35, *p* < .01) (Pindikura et al., 2023). Levels of body esteem were lower in people who were overweight or in the obese weight class compared to those in the ‘normal’ weight category (Waite, Diamond, et al., 2022). Additionally, a further study from Lebanon reported a positive correlation between BMI and body image appreciation (*r* = .29, *p* < .05) (Mahfoud et al., 2023). Other studies reported no significant relationships when looking at associations between BMI and body appearance focus or body satisfaction (Oh et al., 2017), or body attractiveness (Vancampfort et al., 2011). Further linked to weight, people with schizophrenia have been found to hold neutral attitudes towards obesity (Hassamal et al., 2017). People with schizophrenia who were of obese weight class had significantly higher weight stigma (*D* = .289, *p* = .048) compared to people in the non‐obese weight class (Mahfoud et al., 2023). Additionally, BMI may be associated with psychosis symptom content; patients with high BMI were more likely to hear voices saying other people think they are fat (*r* = .421, *p* = .001) and less likely to hear comments saying they were too skinny (Waite et al., 2019) (*r* = −.354, *p* = .007).

###### Metabolic syndrome

One study conducted in Belgium explored the impact of metabolic syndrome and found that patients diagnosed with psychotic conditions with existing glucose abnormalities had lower body image scores after controlling for multiple testing (*p* < .05) (De Hert et al., 2006).

###### Physical exercise capacity and fitness

One study conducted in Belgium reported a moderate negative correlation (*r* = −.40, *p* < .01) between perceived body attractiveness and the amount of distance someone diagnosed with schizophrenia could walk in 6 min (Vancampfort et al., 2011). Another study in people diagnosed with schizophrenia in Poland noted that low evaluation of appearance (*r* = .39; *p* = .001) was correlated with poor evaluation of physical body fitness (Sakson‐Obada et al., 2018).

###### Daily activities

No relationship was observed between body image and time usage in UK‐based people diagnosed with schizophrenia who experienced frequent persecutory delusions (Waite, Diamond, et al., 2022).

###### Insomnia

In people with persecutory delusions, a moderate negative correlation (*r* = −.30, *p* = .001) was observed with insomnia and body esteem (Waite, Diamond, et al., 2022).

##### Other related factors

###### Gender

The evidence for gender differences in body image was mixed. In people with schizophrenia who experience persecutory delusions, there were significant differences between male (*M* = 34.25, *SD* = 15.73) and female (*M* = 24.08, *SD* = 15.11) participants in total body esteem [*t*(113) = 3.45, *p* = .001]. Significant differences were also noted in the appearance [*t*(110) = 3.37, *p* = .001] and weight [*t*(110) = 4.04, *p* < .001] subscales, but not the judgements people make about appearance [*t*(105) = 0.45, *p* = .66]. Females reported lower levels (Waite, Diamond, et al., 2022). In another study of inpatients diagnosed with schizophrenia, there was worse satisfaction with appearance in females compared to males (*M* female = 2.8, *M* male = 1.9; *t* = 4.3, *p* = .001) (Sakson‐Obada et al., 2018). A further study that looked at how much focus people place on appearance found females with schizophrenia were more appearance focused than males with schizophrenia (*t* = −2.41, *p* = .017) (Oh et al., 2017). In a further Australian study, female and male participants diagnosed with a range of psychotic conditions did not show a significant difference between their perceived and desired body size (Tham et al., 2007). One study conducted in a long‐stay forensic ward in New Zealand noted no differences between men and women when asking people if they worried about their body shape, worried about others judging them about their shape or feeling self‐conscious about other people (Every‐Palmer et al., 2018).

###### Quality of life and well‐being

In two UK‐based studies, body image was positively associated with quality of life with a small effect size (*r* = .22, *p* < .01) (Waite, Diamond, et al., 2022). Additionally, people who reported hearing voices that gave positive comments on their body showed a significant correlation with well‐being (*r* = .45, *p* < .001) (Waite et al., 2019).

###### Encounters with the psychiatric system

Neither satisfaction with fitness nor satisfaction with appearance was related to the duration of illness, the number of relapses or the number of psychiatric hospitalisations (Sakson‐Obada et al., 2018) in a study conducted in Poland.

###### Comparisons with controls

Six studies compared people with psychosis spectrum conditions to the general population: four studies report higher rates of body dissatisfaction or body image concerns in people with a diagnosis. Compared to controls matched with respect to age, gender, ethnicity and being a member of a weight management program, people diagnosed with schizophrenia in the USA who were in the obese weight class were less satisfied with weight (*t* = −2.3, *p* < .05) and their shape (*t* = −2.4, *p* < 0. 05) but not appearance (Lundgren et al., 2014). One UK study showed a large difference of lower body image between people experiencing persecutory delusions and non‐matched controls (Waite, Diamond, et al., 2022) for body esteem (*D* = −1.21, *p* < .001), appearance (*D* = −1.11, *p* < .001), weight (*D* = −0.71, *p* < .001) and attribution (*D* = −1.14, *p* < .001). A Belgian study using controls matched for age and gender noted the controls had higher perceived body attractiveness (*p* < .001), perception of sports competence (*p* < .001), physical strength (*p* = .02) and physical self‐worth (*p* < .001) (Vancampfort et al., 2011). (Sakson‐Obada et al., 2018). In a first episode psychosis population conducted in Hong Kong, there were stated to be significant differences in body dissatisfaction between both female and male controls (no *p* value reported) (Wong et al., 2009). Another study conducted in Poland with age and sex matched controls reported no significant differences in satisfaction with body appearance or satisfaction with physical fitness (Sakson‐Obada et al., 2018). No differences in body image dissatisfaction were observed between people with schizophrenia and non‐matched controls in a further Japanese study (Koide et al., 2002). In a single study comparing people with a psychotic condition to patients experiencing a mood disorder without psychosis in the USA, there was no significant difference in body image discrepancy, or attitudes towards obesity (Hassamal et al., 2017).

### Section 3—Qualitative findings

#### Body image from the perspective of people living with psychotic disorders

A further aim of this study was to explore body image from the perspective of people diagnosed with psychotic disorders. Six studies were identified (including the mixed‐methods study) that referred to mostly negative body image. Three key themes and their associated subthemes were constructed and are reported in Table 5. These were then represented in Figure 2 as a framework to guide future research. The supporting qualitative evidence from the manuscripts is now reported.

**TABLE 5:** Meta‐synthesis results.

Main theme	Sub themes
The development of negative body image in psychosis	Internalisation of cultural and social network driven body appearance norms and feeling ‘different’ (Amatullah et al., 2020; Marshall et al., 2020) Memories of appearance‐related trauma and abuse—learning appearance‐related appraisals from others can be unsafe and harmful (Amatullah et al., 2020; Marshall et al., 2020; Waite, Langman, et al., 2022) Psychosis stigma (Becker et al., 2022; Marshall et al., 2020; Waite, Langman, et al., 2022; White et al., 2021) Disempowerment from appearance changes (Every‐Palmer et al., 2018; Marshall et al., 2020; Waite, Langman, et al., 2022; White et al., 2021) Negative body concept (Every‐Palmer et al., 2018; Marshall et al., 2020; Waite, Langman, et al., 2022)
Appearance as a source of threat	Hypervigilance to appearance‐related social input (Marshall et al., 2020; Waite, Langman, et al., 2022; White et al., 2021) Using the body to feel safer (Every‐Palmer et al., 2018; Marshall et al., 2020; Waite, Langman, et al., 2022; White et al., 2021) Feeling unattractive (Marshall et al., 2020; Waite, Langman, et al., 2022, p. 20; White et al., 2021) Becoming body ambivalent (Marshall et al., 2020; Waite, Langman, et al., 2022) Body neglect (Every‐Palmer et al., 2018; Marshall et al., 2020; Waite, Langman, et al., 2022)
Body image goals	Going beyond weight loss—developing positive self‐concept (Marshall et al., 2020; Waite, Langman, et al., 2022) A need for psychosis informed interventions and support (Marshall et al., 2020; Waite, Langman, et al., 2022)

##### Theme 1: The development of negative body image

A theme of negative body image in people living with psychosis was constructed from the available data. Subthemes relevant to understanding the development of negative body image in psychosis are now described.

###### Subtheme: Internalisation of cultural and social network‐driven body appearance norms

The power of normative appearance and the internalisation of appearance norms was apparent in the accounts of people living with psychosis. For example, in a study in Chile, patients highlighted the expectation that people maintain ‘a good outward appearance by and being “neat”’ and ‘decent’ (Becker et al., 2022) (Participant “2.2.7”, p. 68). From a study conducted in India (Amatullah et al., 2020), this was further emphasised by accounts of wanting to be accepted and fit in with the microculture of family and peers and broader society too (Marshall et al., 2020) which emphasised the importance of norm internalisation. Throughout the analysis, there was a sense that experiencing psychosis often violated body appearance norms, and people reported feeling different.

Marshall and colleagues (2020) stated that they observed little gender differences but that ‘males tended to talk more emotively about wanting to feel accepted within relationships with friends and family, whereas female participants spoke more about wanting to be accepted within general society, with a strong desire to “fit in”, “belong” and be “normal”’ (author analysis, p. 648).

Participants were strongly aware of cultural norms about looking well‐being associated with having good health and being accepted by others. This has relevance for patient interactions with mental health professionals and others in their social network. For example, in this quote from a UK‐based participant, they reflect how their recovery from psychosis is judged in part by others from their physical appearance:

If I'm like not well or well to other people, they can read what I'm like. (Robert, p. 651) (Marshall et al., 2020)

###### Subtheme: Memories of appearance‐related trauma and abuse

The meta‐synthesis revealed traumatic memories of appearance‐based bullying and abuse taught people that their body was not safe from others. Participants related this process to their current experiences of negative body image:

I was brought up by a step‐dad, and he basically said to me every day, ‘you're ugly you are, you'll never get a wife’. (John, p. 643) (Marshall et al., 2020)

My mum and dad didn't have much money, I had some jumble sale stuff sometimes and it was embarrassing. And that triggered me off going down the street with people looking at me. (Mandy, p. 646) (Marshall et al., 2020)

This appearance‐related bullying and abuse could also relate to appearance‐based discrimination for protected characteristics such as religion or race.

They would sometimes make fun of it [Sikh Turban]. They would say tomato on your head something like that and I did not have strength to answer, I didn't know myself or my religion to give them valid arguments so it was basically like being weak on the inside and then someone coming and egging your face and then you feel more weak. (Z, p. 353) (Amatullah et al., 2020)

###### Subtheme: Psychosis stigma

Reflecting across a series of interview data, the author highlighted that the development of negative body image was shaped by psychosis stigma, which was additive to other types of discrimination people could experience, such as fat shaming.

Fear of negative evaluation from others was often based on experiences of stigma and fat shaming. (Author Analysis, p. 533) (Waite, Langman, et al., 2022, p. 20)

###### Subtheme: Disempowerment from appearance changes

Participants spoke about their body image in relation to externally imposed changes commonly attributed to antipsychotic weight gain and their strong emotional responses to the same with participants describing devastation and anger. These described a sense of the medication‐related appearance changes being externally imposed.

I'm angry with myself that I'm overweight. I don't like being overweight. (John, p. 647) (Marshall et al., 2020)

Well the whole situation…of being mentally ill and then being made overweight… then having medicine that made me feel ill and overwhelmingly not quite myself, not myself at all. It really devastated me, it changed my life. (Participant 2, p. 532) (Waite, Langman, et al., 2022)

###### Subtheme: Negative body concept

However, appearance‐related changes did not fully explain the association with negative body image; there were accounts where people still felt unhappy about their appearance even when they lost weight which emphasises the role of negative body concept. In the example below, a participant remarks how even once they lost antipsychotic‐related weight, they still disliked themselves and by extension, their appearance:

Even when I lost a load of weight… I just don't like myself. (“Mandy”, p. 649) (Marshall et al., 2020)

##### Theme 2: Appearance as a source of threat

Participants described the physical appearance of their body as something that made them vulnerable to threats from others.

###### Subtheme: Hypervigilance to appearance‐related social input

From the accounts, being hypervigilant to the appraisals of other people or social agents such as voices people hear was a key theme from people's accounts. People reported that other people thought that they were disgusting:

I do get kind of anxious around people and I just assume they want me to leave, you know they want to get rid of me. You know that I'm disgusting. (Participant 3, p. 533) (Waite, Langman, et al., 2022)

Those times are hard because people are physically there to judge, and criticise. And even if they don't physically say stuff, I'll worry that they could be. They might not say ‘you're fat’ but I might be worried that they might think I'm fat or ugly. (Percy, p112) (Marshall et al., 2020)

These social input fears extended to voice hearing experiences, with voices described as powerful social agents that know the insecurities people have and provide cruel comments about appearance:

It's because of the voices, I can hear them (…) they just make comments on what I look like (…) they know all your fears and everything like that and all your insecurities and they just play on it. (Echo, p. 646) (Marshall et al., 2020)

###### Subtheme: Using the body to feel safer

People described trying to cope with this hypervigilance in various ways. In this example, a participant based in a long‐stay forensic unit reflected on how being overweight affords protection and possibly makes them less vulnerable to attack:

describing his weight gain as a deliberate strategy related to ‘*needing to protect myself, for advantage over others and to feel good. I wanted to be bigger than my brother*’. (Author analysis and participant quotation—No pseudonym reported, p. 4) (Every‐Palmer et al., 2018)

For others, avoidance was expressed through avoiding or at least minimising social contact with others.

I withdraw if there's a possibility to withdraw. (Hillary, p. 6.46) (Marshall et al., 2020)

###### Subtheme: Feeling unattractive

A further appearance‐related threat was worries about being unattractive. This could be seen when people discussed how they expected to be perceived by other people as a potential romantic partner.

I think no one will want me 'cause of me weight, no one will really look at me […] I were attractive then, now I put a lot of weight on […] maybe that's why I don't go out, I don't know (pause) I'm ashamed how I look. (Michaela, p. 7) (White et al., 2021)

##### Subtheme: Becoming body ambivalent

Participants remarked that in trying to cope with their body image in everyday life, they had developed feelings of resignation or even acceptance, suggesting a combination of positive, negative and neutral appraisals towards their body image, which represented them becoming ambivalent about their body image and prevented them from feeling constantly unhappy about their appearance.

I just (sigh), accept it, try and accept it, you have to, otherwise you're just going to make yourself angry the whole time. (“Echo”, p. 650) (Marshall et al., 2020)

when you're in hell, what you look like doesn't matter. (Lin, p. 648) (Marshall et al., 2020)

I'd rather be fat than crazy. (Participant 1, p. 533) (Waite, Langman, et al., 2022)

###### Subtheme: Body neglect

Participants shared how in the face of appearance‐related threats and worries about the same, they had come to no longer care about their physical appearance because they struggled with imagining a positive future.

When you don't really see a future there's no rationalising looking after your body 'cause what is the point? (Participant 3, p. 534) (Waite, Langman, et al., 2022).

##### Theme 3: Body image goals

Participants identified body image as something that could be improved upon and as a potential intervention target.

###### Subtheme: Developing positive self‐concept

Taking an overall view from study data, the authors conclude that beyond reducing weight, there was a key identification by participants that they wanted to develop a positive self‐concept about their body.

Tackling weight was seen as a key part of recovery. Yet the starting point for addressing excess weight is a diminished sense of self‐worth, self‐control and self‐efficacy. To begin this journey, participants needed to invest in rebuilding their self‐worth. (Author Analysis, p. 534) (Waite, Langman, et al., 2022)

###### Subtheme: A need for psychosis informed interventions and support

From the findings, it was clear that participants wanted those supporting them to improve their body image to be knowledgeable about the realities of psychosis and common treatment side effects, such as weight gain and how hard it was for participants to lose weight in the face of additional challenges. This is exemplified in the author analysis below:

There was a clear desire to reclaim a sense of normality. Tackling weight was seen as a key part of recovery… Participants worked hard to lose weight. Yet they faced additional burdens and hurdles. Participants spoke of the ‘shadow’ of psychosis. The ongoing presence of psychotic experiences such as voices or paranoia made it harder to engage in weight loss strategies, such as exercise. (Author Analysis, p. 534) (Waite, Langman, et al., 2022)

Furthermore, there were examples where mental health services including inpatient sites were described as creating specific barriers to body‐focused interventions, which are important to highlight in this review because the social environment many participants are living in will likely impact how acceptable body image‐focused interventions are likely to be. This could be seen in the author analysis below, where they reflect on how people in the forensic system stated that prison afforded more opportunity to be physically active.

A number of the forensic patients spontaneously volunteered that they had been more active in prison. (Author Analysis, p. 5) (Every‐Palmer et al., 2018)

### Mixed‐methods summary

In this mixed‐methods review, we synthesised quantitative and qualitative research to (i) examine the relationship between body image and psychosis symptoms; by (ii) exploring body image from the lived experience of people with psychotic disorders and by (iii) synthesising correlates. Taken together, the reviewed data suggest there is evidence of lower levels of body image in people diagnosed with psychotic conditions. People with psychosis reported more negative body image compared to people in the ‘general’ population, and this was more pronounced in females. There were potential links with psychosis symptoms, including paranoia and voice hearing. Further evidence from the general population supports associations between poor body image and delusional‐like beliefs and paranoia. The meta‐synthesis demonstrates that participants spoke about how embodying the social identity of someone living with a psychotic condition influenced their body image and how their body was expected to be and was perceived by others. For example, feeling *disempowered* by appearance changes caused by medication made participants report feeling depressed about their appearance, which amplified worries about being negatively judged by others and that their physical appearance made them vulnerable to threat from others. From participants' perspectives, these worries seemed bi‐directional as some participants reported being on the lookout for negative appearance‐related judgements coming from others due to ‘paranoid’ thinking or feeling ashamed about their appearance. These worries caused participants to retreat and try to avoid social interactions to cope. The negative judgements expected by others seemed driven by a ‘double whack’ of being judged for not having a socially normative appearance and being stigmatised for experiencing psychosis. Of further relevance, voice hearing content mirrored these worries, and there were reports of negative comments about one's appearance. Participants shared experiences of appearance‐related bullying and abuse. While internalising sociocultural norms of what a good appearance should be and not meeting them—and this causing distress—was apparent, there were cases when people reported resisting these norms, including a case where having an overweight body was seen as positive by the participant as it afforded them safety from the threat imposed by others. Participants appeared to have developed an ambivalent attitude towards their appearance, to cope with externally imposed body changes such as antipsychotic weight gain and the distress linked to these. Developing a positive self‐concept of the body was an important treatment goal, but the findings state there is a need for interventions to be psychosis informed.

## DISCUSSION

Body image concerns tend to be elevated in people living with psychotic conditions; body image concerns may be associated with psychotic symptoms; and body image concerns are related to wider physical and mental health challenges that face people living with psychosis spectrum conditions, such as depression and having a high BMI. Patients who were in the obese weight class tended to report lower levels of body image. Moreover, patients who reported episodes of binge eating reported lower levels of body image. People with psychotic spectrum conditions experience poor physical health and may die around 10–25 years before those living without serious mental health problems (Firth et al., 2019). Preliminary evidence from this review suggests people with psychosis already experiencing poor health also have lower levels of body image (De Hert et al., 2006; Vancampfort et al., 2011). From the meta‐synthesis, patients appear ambivalent about their poor physical health and see it as an acceptable trade‐off for avoiding psychosis symptoms, though others reported devastation about physical health changes. People with good body image are more likely to do things to take care of their bodies, such as applying sunscreen (Gillen, 2015), which highlights its value as an intervention target in this area. However, there is a need to highlight systemic issues raised in this review that create barriers to good physical health. For example, patients in a long‐stay ward reported there were limited opportunities to engage in health‐related activities, such as engaging in exercise—supporting findings that wards are obesogenic (Faulkner et al., 2009). People experiencing psychosis may struggle with motivation, which means it might be important for clinicians to maximise opportunities for patients to engage in health‐promoting activities such as these (Michie et al., 2013). From the meta‐synthesis, antipsychotic medication and its associated weight gain were associated with negative body image, but people reported they still disliked their appearance even if they lost weight, which may speak to a negative body concept for some that transcends a simplistic focus on weight.

In the 24 papers on people diagnosed with psychotic conditions, 18 different measurement scales were used, which considered various aspects of body image. Only three scales used in psychosis populations reported psychometric properties which were limited to internal consistency. While the new development of body image scales is cautioned against (Kling et al., 2019), there appears to be a need for a scale more specific to people who experience psychosis spectrum conditions, which will allow for more precise measurement of their body image. For example, appraisals such as someone's physical appearance, making them feel safe or unsafe, would be helpful as this seems a key need from the qualitative synthesis which was not featured in the existing measures. Additionally, with the concerns about poor physical health in relation to psychosis, it may be useful to assess less emotional appraisals, such as how healthy or strong someone feels. The main validated item used in schizophrenia spectrum conditions (BWISE) measures psychological adjustment to body weight and general self‐esteem, which misses some key elements identified from the qualitative work. Additionally, several studies relied on paradigms where participants were asked to choose an ‘ideal figure’ from a range of pictorial options, which gives limited insight into key body image appraisals and likely has Eurocentric normative assumptions about body image. Improving measurement would be essential before focusing on intervention development.

Taking a mixed‐methods approach revealed factors directly drawn from the experience of people diagnosed with psychotic conditions, which could form the basis for intervention development. These include traumatic memories of appearance‐related bullying and abuse, which may be targeted by trauma‐focused interventions, and worries about others' appearance‐related judgements during interactions, which may be targeted by social anxiety‐focused CBT (Aunjitsakul et al., 2021). Participants worried about not feeling attractive or desirable to potential partners. Feeling attractive has been identified as an important yet overlooked aspect of positive body image (Grey et al., 2024) and these findings suggest this matters to people who experience psychosis. The review findings highlighted self‐ambivalence, where people felt resigned to their body and held mixed appraisals of their body image, which were not exclusively negative, highlighting another possible maintenance factor for body image concerns. There may be a role in psychosis body image for ‘self‐ambivalence’ (Godwin et al., 2020) which describes self‐conceptualisation where neutral, positive and negative appraisals co‐exist, In relation to the body, this could mean appraisals such as ‘my body is ugly and I need to accept this and get on with it’, which results in uncertainty about how others may perceive their body: ‘I am unsure if others may think I am ugly’ which may result in preoccupation that can lead individuals experiencing self‐ambivalence to be hypervigilant to cues for how others perceive their body, or making up their minds about what others think, to help resolve this uncertainty. This feels particularly relevant in the context of psychosis and presents a theoretical account for how negative body image may be generated and maintained. Social rank theory, when applied to body image (Carter et al., 2021) posits people are not competing to be ‘better’ than others, just to not be inferior to others and wanting to be socially accepted. However, when this does not happen, people can feel shame and tend toward social isolation behaviours, which was a key theme identified during the meta‐synthesis, and likely maintains poor body image.

### Limitations of the review

This review is the first to systematically summarise the heterogeneous existing literature on this topic and used methods such as pre‐registration, formal analysis of mixed‐methods data to develop a detailed understanding of existing literature, independent peer review of the search strategy, patient co‐leadership in design, analysis and write‐up, and independent screening for articles. However, limitations must be acknowledged. Psychosis is a worldwide concern, and we have limited results in the English language, which likely limits the generalisability of the findings. Additionally, we did not consider ‘grey’ literature. The included studies were conducted in specific populations, settings (such as inpatient wards), and time periods, which may restrict the applicability of the results to other contexts and factors such as cultural differences, healthcare systems and socioeconomic conditions may further influence the generalisability of the findings. Moreover, our broad psychosis spectrum inclusion criteria mean we have included people reporting psychosis spectrum experiences that likely have different and heterogeneous psychopathological manifestations, with separate etiological, physio‐pathologic and psychopathological (affective, cognitive, perceptive and behavioural) underpinnings. Furthermore, the heterogeneity of the results prevented us from conducting a meta‐analysis, and most research is cross‐sectional, so the directionality of effects is unknown. When considering the GRADE criteria, the overall quality of available evidence may be weakened by being at high risk of bias (Murad et al., 2023) and more rigorous research is now required.

### Future research and policy implications

This review has identified key research priorities. There is a need for longitudinal research and interventionist causal designs (Brown et al., 2019) with clinical samples. Similar limitations have been noted in reviews of body image in other mental health conditions, such as what is called borderline personality disorder (Wayda‐Zalewska et al., 2021). Intersectionality describes how social group membership comes with differential exposure to resources and marginalisation, including gender, mental health status and physical appearance (Crenshaw, 1989). It is difficult to understand how intersectional factors influence body image in the context of psychosis; there is a need for diverse samples, including more focus on diverse ethnicity, cultures, genders, sexuality and developmental stages, given that body image may intersect with these aspects of identity. Demographic information relevant to understanding body image, such as ethnicity or race, was not frequently reported or considered in analysis, and future research would be enhanced by including this information—in line with recent guidance (National Institute for Health Research, 2020). Of note, there was limited focus on embodied aspects of cognition (Lee et al., 2022) including interception and proprioception (how the body feels), and how this may relate to body image. Moreover, only one study considered how somatic hallucinations and basic symptoms may relate to body image. Future research may wish to consider condition‐specific issues such as body image changes that may occur during manic episodes in bipolar.

The stigma associated with physical appearance and mental health can intersect in ways that compound the marginalisation and psychological distress experienced by people with psychosis. Participants in this review described a ‘double burden’ of stigma: on one hand, not meeting sociocultural ideals of appearance (such as thinness or conventional attractiveness) led to feelings of shame and social exclusion; on the other, their experiences of psychosis (often misunderstood or feared by the public) further contributed to being judged or devalued. These overlapping stigmas may reinforce each other: appearance‐related stigma may be intensified by assumptions linked to mental illness (e.g., that weight gain reflects laziness) while psychosis‐related stigma may be amplified by visible changes associated with treatment (e.g., weight gain due to taking antipsychotics). This intersection can diminish the quality of life (Barber et al., 2011), lead to social withdrawal and pose barriers to recovery. Future policy‐focused interventions should consider how these forms of stigma are experienced together, and how to foster more affirming, stigma‐informed care environments.

There was limited evidence for what might promote positive body image and positive self‐concept, despite it being identified as a desired target in addition to weight loss. Self‐esteem was identified as a positive correlate for body esteem and positive voice content, such as people receiving messages that they were attractive, which was associated with higher well‐being. Future research is needed to understand what positive body image is and how it is understood from the point of view of people who experience psychosis. When planning future research, it should be noted that people living with serious mental health problems can experience weight stigma from across society, including healthcare professionals, which should be critically considered in future work.

## CONCLUSION

Body image in the context of psychosis appears to be a complex phenomenon with cognitive, emotional and behavioural components. The review synthesised quantitative and qualitative research to examine the relationship between body image and psychosis symptoms. It found evidence that people with psychotic conditions may have a more negative body image compared to the general population, especially among females. Psychosis symptoms, such as paranoia and voice hearing, were cross‐sectionally linked to negative body image. Participants reported feeling disempowered by appearance changes caused by medication, leading to depression and concerns about negative judgements from others. This stigma was described as a ‘double whack’ of not meeting social norms of appearance and being stigmatised for psychosis. The review identified gaps in the literature, including the need for more research with diverse samples. It also called for interventions that develop a positive body concept and consider the impact of weight stigma in healthcare and the impact of psychosis, which could be implemented in routine psychosis care. Future research, especially using longitudinal, interventionist causal designs and validated scales with improved content validity and strong patient involvement, is now required to increase our understanding of body image in psychosis to develop needed interventions.

## AUTHOR CONTRIBUTIONS

**Jenna McAllister:** Conceptualization; data curation; formal analysis; writing – review and editing; methodology. **Sophie Allan:** Formal analysis; writing – original draft; writing – review and editing; validation. **Alie Phiri:** Conceptualization; writing – original draft; writing – review and editing; methodology; formal analysis. **Kara Keddie:** Conceptualization; writing – original draft; writing – review and editing; validation. **Tracey McKee:** Methodology; writing – original draft; writing – review and editing; software; data curation. **Leonie Richardson:** Formal analysis; writing – original draft; conceptualization. **Felicity Waite:** Conceptualization; writing – original draft; methodology; writing – review and editing. **Rebekah Carney:** Writing – original draft; validation. **Gillian MacAfee:** Validation. **Andrew Gumley:** Conceptualization; writing – original draft; writing – review and editing; formal analysis. **Stephanie Allan:** Conceptualization; supervision; methodology; writing – original draft; writing – review and editing; formal analysis.

## CONFLICT OF INTEREST STATEMENT

The authors declare no conflicts of interest.

## Supporting information


Appendix A.

Appendix B.

Appendix C.

Data S1.

## Data Availability

The data analysed is within the summary tables of this manuscript.
