# Supplementary material for: The experience of body image in people with psychosis and psychotic‐like experiences: A co‐produced mixed‐methods systematic review and narrative synthesis
Source: Psychol Psychother. 2025 Nov 17;99(1):1–39. doi: 10.1111/papt.70021 (PMC12905526; doi:10.1111/papt.70021)
Supplement: Supplementary file 1 — Appendix A. [file PAPT-99-1-s003.doc]

**The experience of body image in people with psychosis and psychotic-like experiences: a co-produced mixed methods systematic review and narrative synthesis.**

Final search strategies - run on 16^th^ March 2023, updated 16^th^ January 2024.

- [Ovid MEDLINE(R) ALL <1946 to March 15, 2023>](#_Ovid_MEDLINE(R)_ALL)
- [OVID APA PsycInfo <1806 to March Week 1 2023>](#_APA_PsycInfo_<1806)
- [OVID Embase Classic+Embase <1947 to 2023 March 15>](#_Embase_Classic+Embase_<1947)
- [Cochrane Central Register of Controlled Trials (CENTRAL)](#_Cochrane_Library_(Trials)
- [EBSCOhost CINAHL 1981 to current](#_EBSCOHOST_Cinahl)

## Ovid MEDLINE(R) ALL <1946 to March 15, 2023>

1 schizophrenia/ or schizophrenia, catatonic/ or schizophrenia, disorganized/ or schizophrenia, paranoid/ or schizophrenia, treatment-resistant/ or shared paranoid disorder/ 114307

2 psychotic disorders/ or psychoses, substance-induced/ or psychoses, alcoholic/ 57916

3 Paranoid Disorders/ 4249

4 Affective Disorders, Psychotic/ 2314

5 "Schizophrenia Spectrum and Other Psychotic Disorders"/ 1

6 Hallucinations/ 11822

7 Delusions/ 8109

8 Paranoid Behavior/ 159

9 Hearing/ and Voice/ 181

10 Schizophrenic Psychology/ 34375

11 Bipolar Disorder/ 44779

12 (schizo* or psychosis or psychotic or psychotic-like or paranoi* or (voice* adj3 hear*) or hallucinat* or delusion* or manic depress* or bipolar).ti,ab,kf. 280938

13 1 or 2 or 3 or 4 or 5 or 6 or 7 or 8 or 9 or 10 or 11 or 12 329850

14 body image/ or body dissatisfaction/ or Body Dysmorphic Disorders/ or Physical Appearance, Body/ 20641

15 ((body or bodily) adj3 (image* or regard or perception* or dissatisf* or schema* or satisf* or attractiveness or unattractiveness or self-evaluati* or evaluati* or appear* or esteem or concern* or change* or conscious* or dysmorphi*)).ti,ab,kf. 60955

16 (appearance or self-conscious* or body-conscious* or dysmorphophobia).ti,ab,kf. 252294

17 14 or 15 or 16 316942

18 13 and 17 3522

19 limit 18 to english language 2902

20 Animals/ 7247841

21 Humans/ 21121256

22 20 and 21 2179151

23 20 not 22 5068690

24 19 not 23 2518

## APA PsycInfo <1806 to March Week 1 2023>

1 schizophrenia/ or acute schizophrenia/ or catatonic schizophrenia/ or paranoid schizophrenia/ or process schizophrenia/ or schizoaffective disorder/ or "schizophrenia (disorganized type)"/ or schizophreniform disorder/ or undifferentiated schizophrenia/ 97243

2 psychosis/ or acute psychosis/ or affective psychosis/ or alcoholic psychosis/ or chronic psychosis/ or experimental psychosis/ or "paranoia (psychosis)"/ or postpartum psychosis/ or reactive psychosis/ 33385

3 hallucinations/ or auditory hallucinations/ or visual hallucinations/ 6972

4 delusions/ 5954

5 paranoia/ 1434

6 bipolar disorder/ or bipolar i disorder/ or bipolar ii disorder/ 30231

7 (schizo* or psychosis or psychotic or psychotic-like or paranoi* or (voice* adj3 hear*) or hallucinat* or delusion* or manic depress* or bipolar).ti,ab,id. 235370

8 1 or 2 or 3 or 4 or 5 or 6 or 7 237727

9 body image/ or body esteem/ or body image disturbances/ 14370

10 body dysmorphic disorder/ 1340

11 ((body or bodily) adj3 (image* or regard or perception* or dissatisf* or schema* or satisf* or attractiveness or unattractiveness or self-evaluati* or evaluati* or appear* or esteem or concern* or change* or conscious* or dysmorphi*)).ti,ab,id. 30015

12 (appearance or self-conscious* or body-conscious* or dysmorphophobia).ti,ab,id. 35885

13 9 or 10 or 11 or 12 63111

14 8 and 13 2936

15 limit 14 to english language 2273

16 animal.po. 435699

17 human.po. 4505446

18 16 and 17 52479

19 16 not 18 383220

20 15 not 19 2211

## Embase Classic+Embase <1947 to 2023 March 15>

1 schizophrenia/ or catatonic schizophrenia/ or hebephrenia/ or latent schizophrenia/ or negative syndrome/ or paranoid schizophrenia/ or positive syndrome/ or residual schizophrenia/ or schizophrenic reaction/ or simple schizophrenia/ or treatment-resistant schizophrenia/ 220869

2 psychosis/ or acute psychosis/ or affective psychosis/ or alcohol psychosis/ or brief psychotic disorder/ or drug induced psychosis/ or manic psychosis/ or paranoid psychosis/ or puerperal psychosis/ 126710

3 paranoia/ 13155

4 hallucination/ or auditory hallucination/ or visual hallucination/ 44678

5 delusion/ or grandiose delusion/ or persecutory delusion/ 21590

6 bipolar disorder/ or bipolar i disorder/ or bipolar ii disorder/ 73924

7 (schizo* or psychosis or psychotic or psychotic-like or paranoi* or (voice* adj3 hear*) or hallucinat* or delusion* or manic depress* or bipolar).ti,ab,kf. 408276

8 1 or 2 or 3 or 4 or 5 or 6 or 7 510100

9 body image/ or body dissatisfaction/ 27770

10 body dysmorphic disorder/ 3605

11 physical appearance/ 2636

12 ((body or bodily) adj3 (image* or regard or perception* or dissatisf* or schema* or satisf* or attractiveness or unattractiveness or self-evaluati* or evaluati* or appear* or esteem or concern* or change* or conscious* or dysmorphi*)).ti,ab,kf. 86331

13 (appearance or self-conscious* or body-conscious* or dysmorphophobia).ti,ab,kf. 362096

14 9 or 10 or 11 or 12 or 13 452510

15 8 and 14 6388

16 limit 15 to english language 5244

17 animal/ 2092408

18 human/ 26422308

19 17 and 18 506506

20 17 not 19 1585902

21 16 not 20 5176

## Cochrane Library (Trials only)

Date Run: 16/03/2023 12:55:35

Comment:

ID Search Hits

#1 MeSH descriptor: [Schizophrenia] explode all trees 8995

#2 MeSH descriptor: [Psychotic Disorders] this term only 3489

#3 MeSH descriptor: [Psychoses, Substance-Induced] explode all trees 211

#4 MeSH descriptor: [Paranoid Disorders] this term only 113

#5 MeSH descriptor: [Affective Disorders, Psychotic] this term only 105

#6 MeSH descriptor: [Schizophrenia Spectrum and Other Psychotic Disorders] this term only 34

#7 MeSH descriptor: [Hallucinations] this term only 430

#8 MeSH descriptor: [Delusions] this term only 201

#9 MeSH descriptor: [Paranoid Behavior] this term only 6

#10 MeSH descriptor: [Hearing] this term only 413

#11 MeSH descriptor: [Voice] this term only 206

#12 #10 AND #11 1

#13 MeSH descriptor: [Schizophrenic Psychology] this term only 2397

#14 MeSH descriptor: [Bipolar Disorder] this term only 3261

#15 ((schizo* or psychosis or psychotic or psychotic-like or paranoi* or (voice* NEAR/2 hear*) or hallucinat* or delusion* or manic depress* or bipolar)):ti,ab,kw (Word variations have been searched) 35225

#16 #1 OR #2 OR #3 OR #4 OR #5 OR #6 OR #7 OR #8 OR #9 OR #12 OR #13 OR #14 OR #15 35302

#17 MeSH descriptor: [Body Image] explode all trees 908

#18 MeSH descriptor: [Body Dysmorphic Disorders] this term only 92

#19 MeSH descriptor: [Physical Appearance, Body] this term only 9

#20 (((body or bodily) NEAR/2 (image* or regard or perception* or dissatisf* or schema* or satisf* or attractiveness or unattractiveness or self-evaluati* or evaluati* or appear* or esteem or concern* or change* or conscious* or dysmorphi*))):ti,ab,kw (Word variations have been searched) 10930

#21 ((appearance or self-conscious* or body-conscious* or dysmorphophobia)):ti,ab,kw (Word variations have been searched) 80931

#22 #17 OR #18 OR #19 OR #20 OR #21 90865

#23 #16 AND #22 in Trials 2124

## EBSCOhost CINAHL

Top of Form

| **#** | **Query** | **Limiters/Expanders** | **Last Run Via** | **Results** |
| --- | --- | --- | --- | --- |
| S19 | s14 NOT s18 | Limiters - English Language Search modes - Boolean/Phrase | Interface - EBSCOhost Research Databases Search Screen - Advanced Search Database - CINAHL | 529 |
| S18 | s15 NOT s17 | Limiters - English Language Search modes - Boolean/Phrase | Interface - EBSCOhost Research Databases Search Screen - Advanced Search Database - CINAHL | 88,660 |
| S17 | s15 AND s16 | Limiters - English Language Search modes - Boolean/Phrase | Interface - EBSCOhost Research Databases Search Screen - Advanced Search Database - CINAHL | 8,324 |
| S16 | (MH "Human") | Limiters - English Language Search modes - Boolean/Phrase | Interface - EBSCOhost Research Databases Search Screen - Advanced Search Database - CINAHL | 2,564,046 |
| S15 | (MH "Animals") | Limiters - English Language Search modes - Boolean/Phrase | Interface - EBSCOhost Research Databases Search Screen - Advanced Search Database - CINAHL | 96,984 |
| S14 | S7 AND S12 | Limiters - English Language Search modes - Boolean/Phrase | Interface - EBSCOhost Research Databases Search Screen - Advanced Search Database - CINAHL | 535 |
| S13 | S7 AND S12 | Search modes - Boolean/Phrase | Interface - EBSCOhost Research Databases Search Screen - Advanced Search Database - CINAHL | 555 |
| S12 | S8 OR S9 OR S10 OR S11 | Search modes - Boolean/Phrase | Interface - EBSCOhost Research Databases Search Screen - Advanced Search Database - CINAHL | 55,101 |
| S11 | TI ( (appearance or self-conscious* or body-conscious* or dysmorphophobia ) OR AB ( (appearance or self-conscious* or body-conscious* or dysmorphophobia ) | Search modes - Boolean/Phrase | Interface - EBSCOhost Research Databases Search Screen - Advanced Search Database - CINAHL | 30,540 |
| S10 | TI ( ((body or bodily) N2 (image* or regard or perception* or dissatisf* or schema* or satisf* or attractiveness or unattractiveness or self-evaluati* or evaluati* or appear* or esteem or concern* or change* or conscious* or dysmorphi*)) ) OR AB ( ((body or bodily) N2 (image* or regard or perception* or dissatisf* or schema* or satisf* or attractiveness or unattractiveness or self-evaluati* or evaluati* or appear* or esteem or concern* or change* or conscious* or dysmorphi*)) ) | Search modes - Boolean/Phrase | Interface - EBSCOhost Research Databases Search Screen - Advanced Search Database - CINAHL | 21,171 |
| S9 | (MH "Body Dysmorphic Disorder") | Search modes - Boolean/Phrase | Interface - EBSCOhost Research Databases Search Screen - Advanced Search Database - CINAHL | 787 |
| S8 | (MH "Body Image") OR (MH "Body Dissatisfaction") | Search modes - Boolean/Phrase | Interface - EBSCOhost Research Databases Search Screen - Advanced Search Database - CINAHL | 13,173 |
| S7 | S1 OR S2 OR S3 OR S4 OR S5 OR S6 | Search modes - Boolean/Phrase | Interface - EBSCOhost Research Databases Search Screen - Advanced Search Database - CINAHL | 78,624 |
| S6 | TI ( (schizo* or psychosis or psychotic or psychotic-like or paranoi* or (voice* N2 hear*) or hallucinat* or delusion* or manic depress* or bipolar) ) OR AB ( (schizo* or psychosis or psychotic or psychotic-like or paranoi* or (voice* N2 hear*) or hallucinat* or delusion* or manic depress* or bipolar) ) | Search modes - Boolean/Phrase | Interface - EBSCOhost Research Databases Search Screen - Advanced Search Database - CINAHL | 66,488 |
| S5 | (MH "Delusions") | Search modes - Boolean/Phrase | Interface - EBSCOhost Research Databases Search Screen - Advanced Search Database - CINAHL | 1,966 |
| S4 | (MH "Hallucinations") | Search modes - Boolean/Phrase | Interface - EBSCOhost Research Databases Search Screen - Advanced Search Database - CINAHL | 3,444 |
| S3 | (MH "Psychoses, Substance-Induced") OR (MH "Psychoses, Alcoholic") | Search modes - Boolean/Phrase | Interface - EBSCOhost Research Databases Search Screen - Advanced Search Database - CINAHL | 543 |
| S2 | (MH "Psychotic Disorders") OR (MH "Affective Disorders, Psychotic") OR (MH "Bipolar Disorder") OR (MH "Postpartum Psychosis") OR (MH "Schizoaffective Disorder") OR (MH "Paranoid Disorders") | Search modes - Boolean/Phrase | Interface - EBSCOhost Research Databases Search Screen - Advanced Search Database - CINAHL | 28,469 |
| S1 | (MH "Schizophrenia, Treatment-Resistant") OR (MH "Schizophrenia") | Search modes - Boolean/Phrase | Interface - EBSCOhost Research Databases Search Screen - Advanced Search Database - CINAHL | 28,337 |

Bottom of Form
