# Supplementary material for: The experience of body image in people with psychosis and psychotic‐like experiences: A co‐produced mixed‐methods systematic review and narrative synthesis
Source: Psychol Psychother. 2025 Nov 17;99(1):1–39. doi: 10.1111/papt.70021 (PMC12905526; doi:10.1111/papt.70021)
Supplement: Supplementary file 2 — Appendix B. [file PAPT-99-1-s002.docx]

| Study Name | Screening 1- Are there clear research questions? | Screening 2 - Do the collected data allow to address the research questions? | Qualitative 1 - Is the qualitative approach appropriate to answer the research question? | Qualitative 2- Are the qualitative data collection methods adequate to address the research question? | Qualitative 3 - Are the findings adequately derived from the data? | Qualitative 4 - Is the interpretation of results sufficiently substantiated by data? | Qualitative 5 - Is the interpretation of results sufficiently substantiated by data? | Quantitative non RCT 1 - Are the participants representative of the target population? | Quantitative non RCT 2 - Are measurements appropriate regarding both the outcome and intervention (or exposure)? | Quantitative non RCT 3 - Are there complete outcome data? | Quantitative non RCT 4 - Are the confounders accounted for in the design and analysis? | Quantitative non RCT 5 - During the study period, is the intervention administered (or exposure occurred) as intended? | Quantitative Descriptive 1 Is the sampling strategy relevant to address the research question? | Quantitative Descriptive 2 Is the sample representative of the target population? | Quantitative Descriptive 3 Are the measurements appropriate? | Quantitative Descriptive 4 Is the risk of nonresponse bias low? | Quantitative Descriptive 5 Is the statistical analysis appropriate to answer the research question? | Mixed Methods - 1 Is there an adequate rationale for using a mixed methods design to address the research question? | Mixed Methods - 2 Are the different components of the study effectively integrated to answer the research question? | Mixed Methods - 3 Are the outputs of the integration of qualitative and quantitative components adequately interpreted? | Mixed Methods - 4 Are divergences and inconsistencies between quantitative and qualitative results adequately addressed? | Mixed Methods - 5 Do the different components of the study adhere to the quality criteria of each tradition of the methods involved? |
| --- | --- | --- | --- | --- | --- | --- | --- | --- | --- | --- | --- | --- | --- | --- | --- | --- | --- | --- | --- | --- | --- | --- |
| Al Habibi 2012 | 1 | 1 |  |  |  |  |  |  |  |  |  |  | 1 | 0 | 1 | 0 | 1 |  |  |  |  |  |
| Amatullah 2020 | 1 | 1 | 1 | 1 | 1 | 1 | 1 |  |  |  |  |  |  |  |  |  |  |  |  |  |  |  |
| Bagrowska 2022 | 1 | 1 |  |  |  |  |  |  |  |  |  |  | 1 | 0 | 1 | 0 | 1 |  |  |  |  |  |
| Becker 2022 | 1 | 1 | 1 | 1 | 1 | 0 | 1 |  |  |  |  |  |  |  |  |  |  |  |  |  |  |  |
| De Hert 2006 | 1 | 1 |  |  |  |  |  |  |  |  |  |  | 1 | 0 | 1 | 0 | 1 |  |  |  |  |  |
| Dikmen 2022 | 1 | 1 |  |  |  |  |  |  |  |  |  |  | 1 | 0 | 1 | 0 | 1 |  |  |  |  |  |
| EveryPalmer_2018 | 1 | 1 |  |  |  |  |  |  |  |  |  |  |  |  |  |  |  | 1 | 1 | 1 | 0 | 0 |
| Fekih-Romdhane 2023 | 1 | 1 |  |  |  |  |  | 0 | 1 | 0 | 1 | 1 |  |  |  |  |  |  |  |  |  |  |
| Goyal 2019 | 1 | 1 |  |  |  |  |  |  |  |  |  |  | 1 | 0 | 0 | 0 | 1 |  |  |  |  |  |
| Hassamal 2017 | 1 | 1 |  |  |  |  |  |  |  |  |  |  | 1 | 0 | 0 | 0 | 1 |  |  |  |  |  |
| Keating 2016 | 1 | 1 |  |  |  |  |  |  |  |  |  |  | 1 | 0 | 1 | 0 | 1 |  |  |  |  |  |
| Koide 2002 | 1 | 1 |  |  |  |  |  |  |  |  |  |  | 1 | 0 | 0 | 0 | 1 |  |  |  |  |  |
| Lavell 2014 | 1 | 1 |  |  |  |  |  |  |  |  |  |  | 1 | 0 | 1 | 0 | 1 |  |  |  |  |  |
| Lee 2021 | 1 | 1 |  |  |  |  |  |  |  |  |  |  | 1 | 0 | 1 | 0 | 1 |  |  |  |  |  |
| Lundgren 2014 | 1 | 1 |  |  |  |  |  |  |  |  |  |  | 1 | 0 | 1 | 0 | 1 |  |  |  |  |  |
| Mahfoud 2023 | 1 | 1 |  |  |  |  |  |  |  |  |  |  | 1 | 1 | 1 | 1 | 1 |  |  |  |  |  |
| Malcolm 2022 | 1 | 1 |  |  |  |  |  |  |  |  |  |  | 1 | 0 | 0 | 0 | 1 |  |  |  |  |  |
| Marshall 2020 | 1 | 1 | 1 | 1 | 1 | 1 | 1 |  |  |  |  |  |  |  |  |  |  |  |  |  |  |  |
| Oh 2017 | 1 | 1 |  |  |  |  |  |  |  |  |  |  | 1 | 1 | 1 | 1 | 1 |  |  |  |  |  |
| Pindikdura 2022 | 1 | 1 |  |  |  |  |  |  |  |  |  |  | 1 | 0 | 1 | 0 | 1 |  |  |  |  |  |
| Röhricht 2002 | 1 | 1 |  |  |  |  |  |  |  |  |  |  | 1 | 0 | 0 | 0 | 1 |  |  |  |  |  |
| SaksonObada 2018 | 1 | 1 |  |  |  |  |  |  |  |  |  |  | 1 | 0 | 0 | 0 | 1 |  |  |  |  |  |
| Tham 2007 | 1 | 1 |  |  |  |  |  |  |  |  |  |  | 1 | 0 | 0 | 0 | 1 |  |  |  |  |  |
| Toh 2022 | 1 | 1 |  |  |  |  |  |  |  |  |  |  | 1 | 0 | 1 | 0 | 1 |  |  |  |  |  |
| Vancampfort 2011 | 1 | 1 |  |  |  |  |  |  |  |  |  |  | 1 | 0 | 1 | 1 | 1 |  |  |  |  |  |
| Waite 2017 | 1 | 1 |  |  |  |  |  |  |  |  |  |  | 1 | 1 | 0 | 1 | 1 |  |  |  |  |  |
| Waite 2019 | 1 | 1 |  |  |  |  |  |  |  |  |  |  | 1 | 0 | 1 | 0 | 1 |  |  |  |  |  |
| Waite 2022 (Qualitative) | 1 | 1 | 1 | 1 | 1 | 1 | 1 |  |  |  |  |  |  |  |  |  |  |  |  |  |  |  |
| Waite 2022 (Quantitative) | 1 | 1 |  |  |  |  |  |  |  |  |  |  | 1 | 1 | 1 | 0 | 1 |  |  |  |  |  |
| White 2021 | 1 | 1 | 1 | 1 | 1 | 1 | 1 |  |  |  |  |  |  |  |  |  |  |  |  |  |  |  |
| Wong 2009 | 1 | 1 |  |  |  |  |  |  |  |  |  |  | 1 | 0 | 0 | 0 | 1 |  |  |  |  |  |
