# Supplementary material for: The experience of body image in people with psychosis and psychotic‐like experiences: A co‐produced mixed‐methods systematic review and narrative synthesis
Source: Psychol Psychother. 2025 Nov 17;99(1):1–39. doi: 10.1111/papt.70021 (PMC12905526; doi:10.1111/papt.70021)
Supplement: Supplementary file 3 — Appendix C. [file PAPT-99-1-s001.docx]

Table 1: Control Demographics Typical Population (n = 624)

| Weighted Mean Age | 36.77 |
| --- | --- |
| Weighted Mean Gender (% female) | 49.51 |

Table 2: Control Demographics Non Psychotic Mental Health Conditions (n = 129)

| Weighted Mean Age | 45.77 |
| --- | --- |
| Weighted Mean Gender (% female) | 52 |

Table 2: Typical Population Demographics (n = 17,670)

| Weighted Mean Age | 25.62 |
| --- | --- |
| Weighted Mean Gender (% female) | 53.03 |
