# Supplementary material for: The experience of body image in people with psychosis and psychotic‐like experiences: A co‐produced mixed‐methods systematic review and narrative synthesis
Source: Psychol Psychother. 2025 Nov 17;99(1):1–39. doi: 10.1111/papt.70021 (PMC12905526; doi:10.1111/papt.70021)
Supplement: Supplementary file 4 — Data S1. [file PAPT-99-1-s004.docx]

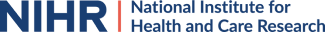


**PROSPERO**

International prospective register of systematic reviews

**Body image concerns in people with psychosis and psychotic like experiences: a mixed methods systematic review**

*Jenna McAllister, Andrew Gumley, Andrew Gumley, Felicity Waite, Tracey McKee,*

*Stephanie Allan*

# **Citation**

Jenna McAllister, Andrew Gumley, Andrew Gumley, Felicity Waite, Tracey McKee, Stephanie Allan. Body image concerns in people with psychosis and psychotic like experiences: a mixed methods systematic review. PROSPERO 2024 Available from <https://www.crd.york.ac.uk/PROSPERO/view/CRD42023407023>

# REVIEW TITLE AND BASIC DETAILS

## Review title

Body image concerns in people with psychosis and psychotic like experiences: a mixed methods systematic review

## Review objectives

What is the lived experience of body image concerns in people with a diagnosis of psychosis or experiencing psychotic experiences?

Do body image concerns and psychotic like experiences co-occur?

Do body image concerns predict onset or persistence for psychotic experiences and vice versa?

Does a change in one lead to a change in the other?

What other correlates have been considered in studies that explore these two concepts?

**Keywords**

Body Appearance, Body image, Psychosis, Self-Concept

# SEARCHING AND SCREENING

## Searches

Articles will be identified through a search according to -best practice guidelines. Databases

OVID MEDLINE (R) ALL, OVID APA PsycINFO, and Cochrane Central Register of Controlled

Trials (CENTRAL), OVID EMBASE Classic + Embase, EBSCOhost CINAHL will be searched from their inception to the present time. A range of subject headings and keywords related to psychosis and body image will be searched in all databases:

The following search algorithm will be used: (“schizo*” OR “schizophrenia” OR “psychosis” OR

“paranoia” OR “voices” OR “hallucination* OR “delusion* OR “psychotic*”) AND (“body image” OR “body regard” OR “body perception” OR “body dissatisfaction” OR “body schema” OR “body satisfaction” OR “body attractiveness” OR “body unattractiveness” OR “body self-evaluation” OR “body evaluation” OR “body appearance”).

The reference lists of studies meeting the inclusion criteria will be searched to identify additional relevant studies and researchers specialising in schizophrenia will be contacted for input. Forward citations will be reviewed using Google Scholar. Studies will be limited to the English language. Two researchers will screen articles for eligibility independently. Basic inclusion criteria will be checked first, and articles meeting the criteria will be retrieved and checked by two researchers (SA and JM) again using PICO criteria. We will report Cohen’s Kappa. In the event of disagreement AG will decide.

Rayyan software will be used to manage the screening process.

## Study design

Following guidance on mixed-methods systematic reviews (Hong, Pluye, Bujold, & Wassef, 2017) we will define qualitative research as: “research that aims at exploring and understanding phenomena in terms of the meanings people bring to the specific phenomenon” and quantitative research as “ research that aims at testing or developing theories by examining the relationship among variables”.

This review will include:

Published qualitative studies about body image concerns and about broader topics that include this particular theme.

Published quantitative studies exploring body image concerns and potential associations with other psychotic-like experiences.

Any mixed-methods study which contains either of the above

# ELIGIBILITY CRITERIA

## Condition or domain being studied

Body image – broadly defined as a body schema or how someone evaluates their own body (Kling et al, 2019).

## Population

People with a diagnosis of affective and non-affective psychosis spectrum conditions (including first episode psychosis).

•Analogue studies where psychotic-like experiences are assessed with continuum-based scales.

**Intervention(s) or exposure(s)** Inclusion:

Qualitative studies – a key focus on body image concerns in studies exploring psychosis or psychotic-like experiences in either aim or findings.

Quantitative studies – should have a measure of body image concerns and at least one psychotic or psychotic-like experience and test the relationship between the two. We will summarise other “key outcomes” such as quality of life.

Mixed methods studies featuring either of the above.

Exclusion:

Studies with no specific focus on body image concerns or psychotic-like experiences.

Studies relating primarily to dementias or other organic psychoses.

**Comparator(s) or control(s)** Not applicable

# OUTCOMES TO BE ANALYSED

## Main outcomes

Quantitative associations between psychotic-like experiences such as paranoid thoughts and body image concerns.

Qualitative themes related to body image concerns and psychosis experiences.

*Measures of effect*

Quantitative associations will include effect sizes.

Qualitative themes will include relevant qualitative data.

## Additional outcomes

We are interested in what has been measured and will include other outcomes such as quality of life, depression, and anxiety.

*Measures of effect*

Quantitative associations will include effect sizes.

Qualitative themes will include relevant qualitative data.

# DATA COLLECTION PROCESS

## Data extraction (selection and coding)

A first extraction spreadsheet will be designed, and piloted and appropriate changes will be made for this review. The form will identify: Name of authors and Year (of publication), countries (in which research was carried out), mean age and standard deviation (of participants), gender, study design, and specific diagnosis (i.e. first episode psychosis) if relevant.

## Risk of bias (quality) assessment

For the quality assessment, two researchers will independently rate papers, and we will report inter-rater agreement. In the event of disagreement, another team member's opinion will be sought. We will use the Mixed Methods critical Appraisal Tool that is designed for the appraisal stage of systematic mixed studies reviews (reviews that include qualitative, quantitative and mixed methods studies). It permits the appraisal of the methodological quality of five categories to studies: qualitative research, randomised controlled trials, non-randomised studies, quantitative descriptive studies, and mixed methods studies (Hong, 2018). This will enable us to make an overall comment on the quality of studies in this area and will be a key outcome of this review. We will adapt the tool slightly to measure whether researchers conducting quantitative research have performed a power calculation.

# PLANNED DATA SYNTHESIS

## Strategy for data synthesis

Our primary approach will be to present a descriptive mixed-methods narrative review of results, supported by tabulated displays of details of included studies; summary of findings and risk of bias. In line with Hong and colleagues, we will employ a results-based convergent synthesis design for this mixed-methods review, where qualitative and quantitative evidence will be analysed separately using different synthesis methods and results of both syntheses are integrated during a final synthesis. We will describe our plan to analyse qualitative data and quantitative data in turn:

For qualitative data, we will use Thomas and Harden’s approach to meta-synthesis (Thomas and Harden, 2008) will be used for data synthesis of the studies. The approach consists of 3 stages: line-by-line coding of findings allows for concepts to be translated from one study to another. establishing descriptive themes in the previously coded findings and developing new codes creating analytical themes

Quantitative Data

We will collect and report statistically significant correlates, covariates, mediators, moderators, and causal analysis that are reported between body image concerns and psychosis or psychotic-like experiences in studies. We will tabulate the results.

Once qualitative and quantitative data have been analysed, data will be synthesised by into a single summary exploring how body image concerns relate to psychotic-like experiences (likely a proposed psychological model) to answer the research questions of this review. Due to the constructed nature of this approach, we will report a reflexive account from all authors and report reasoning for linking any concepts in the proposed model and how this links to our earlier quantitative and qualitative analysis. We will report our approach in line with SWIM guidelines (Campbell, 2020). Following the lead author’s first analysis, all other authors will critique the final synthesis, and we will use group consensus to construct the model. We will report this transparently if we cannot agree on any aspect.

**Analysis of subgroups or subsets** Not applicable

# REVIEW AFFILIATION, FUNDING AND PEER REVIEW

## Review team members

Ms Jenna McAllister, University of Glasgow

Professor Andrew Gumley, University of Glasgow

Professor Andrew Gumley, University of Glasgow

Dr Felicity Waite, University of Oxford

Ms Tracey McKee, NHS Greater Glasgow & Clyde Ms Stephanie Allan, University of Glasgow

**Review affiliation**

University of Glasgow

**Funding source**

Not applicable

## Named contact

Stephanie Allan. Clarice Pears Building, University Of Glasgow, Byres Rd, Glasgow G12 8TA stephanie.allan@glasgow.ac.uk

# TIMELINE OF THE REVIEW

**Review timeline**

Start date: 10 March 2023. End date: 30 April 2024

**Date of first submission to PROSPERO** 10 March 2023

**Date of registration in PROSPERO** 10 March 2023

# CURRENT REVIEW STAGE

**Publication of review results**

The intention is to publish the review once completed.The review will be published in English

## Stage of the review at this submission

**Review stage Started Completed**

Pilot work

Formal searching/study identification

Screening search results against inclusion criteria

Data extraction or receipt of IP

Risk of bias/quality assessment

Data synthesis

**Review status**

The review is currently planned or ongoing.

# ADDITIONAL INFORMATION

## PROSPERO version history

Version 1.0 published on 10 Mar 2023

**Review conflict of interest** None known

**Country**

England, Scotland

**Medical Subject Headings**

Body Image; Humans; Psychotic Disorders

## Disclaimer

The content of this record displays the information provided by the review team. PROSPERO does not peer review registration records or endorse their content.

PROSPERO accepts and posts the information provided in good faith; responsibility for record content rests with the review team. The owner of this record has affirmed that the information provided is truthful and that they understand that deliberate provision of inaccurate information may be construed as scientific misconduct.

PROSPERO does not accept any liability for the content provided in this record or for its use.

Readers use the information provided in this record at their own risk.

Any enquiries about the record should be referred to the named review contact
